# Tackling Rapid Radiations With Targeted Sequencing

**DOI:** 10.3389/fpls.2019.01655

**Published:** 2020-01-09

**Authors:** Isabel Larridon, Tamara Villaverde, Alexandre R. Zuntini, Lisa Pokorny, Grace E. Brewer, Niroshini Epitawalage, Isabel Fairlie, Marlene Hahn, Jan Kim, Enrique Maguilla, Olivier Maurin, Martin Xanthos, Andrew L. Hipp, Félix Forest, William J. Baker

**Affiliations:** ^1^Royal Botanic Gardens, Kew, Surrey, United Kingdom; ^2^Systematic and Evolutionary Botany Lab, Department of Biology, Ghent University, Ghent, Belgium; ^3^Real Jardín Botánico (RJB-CSIC), Madrid, Spain; ^4^The Morton Arboretum, Lisle, IL, United States; ^5^The Field Museum, Chicago, IL, United States; ^6^Centre for Plant Biotechnology and Genomics (CBGP, UPM-INIA), Madrid, Spain; ^7^Department of Animal and Plant Sciences, University of Sheffield, Sheffield, United Kingdom; ^8^Departamento de Biología Vegetal y Ecología, Universidad de Sevilla, Sevilla, Spain

**Keywords:** C4 *Cyperus* clade, Cyperaceae, Plant and Fungal Trees of Life, phylogenomics, polytomy, targeted sequencing

## Abstract

In phylogenetic studies across angiosperms, at various taxonomic levels, polytomies have persisted despite efforts to resolve them by increasing sampling of taxa and loci. The large amount of genomic data now available and statistical tools to analyze them provide unprecedented power for phylogenetic inference. Targeted sequencing has emerged as a strong tool for estimating species trees in the face of rapid radiations, lineage sorting, and introgression. Evolutionary relationships in Cyperaceae have been studied mostly using Sanger sequencing until recently. Despite ample taxon sampling, relationships in many genera remain poorly understood, hampered by diversification rates that outpace mutation rates in the loci used. The C4 *Cyperus* clade of the genus *Cyperus* has been particularly difficult to resolve. Previous studies based on a limited set of markers resolved relationships among *Cyperus* species using the C3 photosynthetic pathway, but not among C4 *Cyperus* clade taxa. We test the ability of two targeted sequencing kits to resolve relationships in the C4 *Cyperus* clade, the universal Angiosperms-353 kit and a Cyperaceae-specific kit. Sequences of the targeted loci were recovered from data generated with both kits and used to investigate overlap in data between kits and relative efficiency of the general and custom approaches. The power to resolve shallow-level relationships was tested using a summary species tree method and a concatenated maximum likelihood approach. High resolution and support are obtained using both approaches, but high levels of missing data disproportionately impact the latter. Targeted sequencing provides new insights into the evolution of morphology in the C4 *Cyperus* clade, demonstrating for example that the former segregate genus *Alinula* is polyphyletic despite its seeming morphological integrity. An unexpected result is that the *Cyperus margaritaceus*-*Cyperus niveus* complex comprises a clade separate from and sister to the core C4 *Cyperus* clade. Our results demonstrate that data generated with a family-specific kit do not necessarily have more power than those obtained with a universal kit, but that data generated with different targeted sequencing kits can often be merged for downstream analyses. Moreover, our study contributes to the growing consensus that targeted sequencing data are a powerful tool in resolving rapid radiations.

## Introduction

Since the late 1980s, molecular phylogenetics has yielded major new insights into the evolution of land plants, especially for flowering plants (e.g., Chase et al., 1993; Ruhfel et al., 2014; Wickett et al., 2014; APG IV, 2016). However, uncertainty in topologies has persisted, particularly for deep nodes (Wickett et al., 2014) and for ancient and recent rapid radiations, which are often inferred as polytomies (Fishbein et al., 2001; Whitfield and Lockhart, 2007; Snak et al., 2016; Spalink et al., 2016). Researchers have attempted to resolve these issues by increasing taxon sampling, the number of DNA loci sampled, or both (e.g., Philippe et al., 2011; Nabhan and Sarkar, 2012; Nicholls et al., 2015).

Targeted sequencing of genomic libraries can yield hundreds to thousands of DNA loci across multiple individuals and species, depending on the targeted sequencing kit used (e.g., Faircloth et al., 2018; Johnson et al., 2018; Couvreur et al., 2019), providing sequencing data suitable to addressing challenging and outstanding problems in plant systematics. It is an extremely versatile technique that can be used to solve ancient and recent species radiations (Nicholls et al., 2015; Stevens et al., 2015; Mitchell et al., 2017; Kadlec et al., 2017), as well as to bridge micro- and macroevolutionary levels (Kates et al., 2018; Villaverde et al., 2018). Additionally, it works well with degraded DNA template, e.g., herbarium material (Hart et al., 2016; McKain et al., 2018; Brewer et al., 2019). Targeted sequencing is rapidly becoming a standard phylogenomic method for flowering plants (McKain et al., 2018).

**Figure 1:**
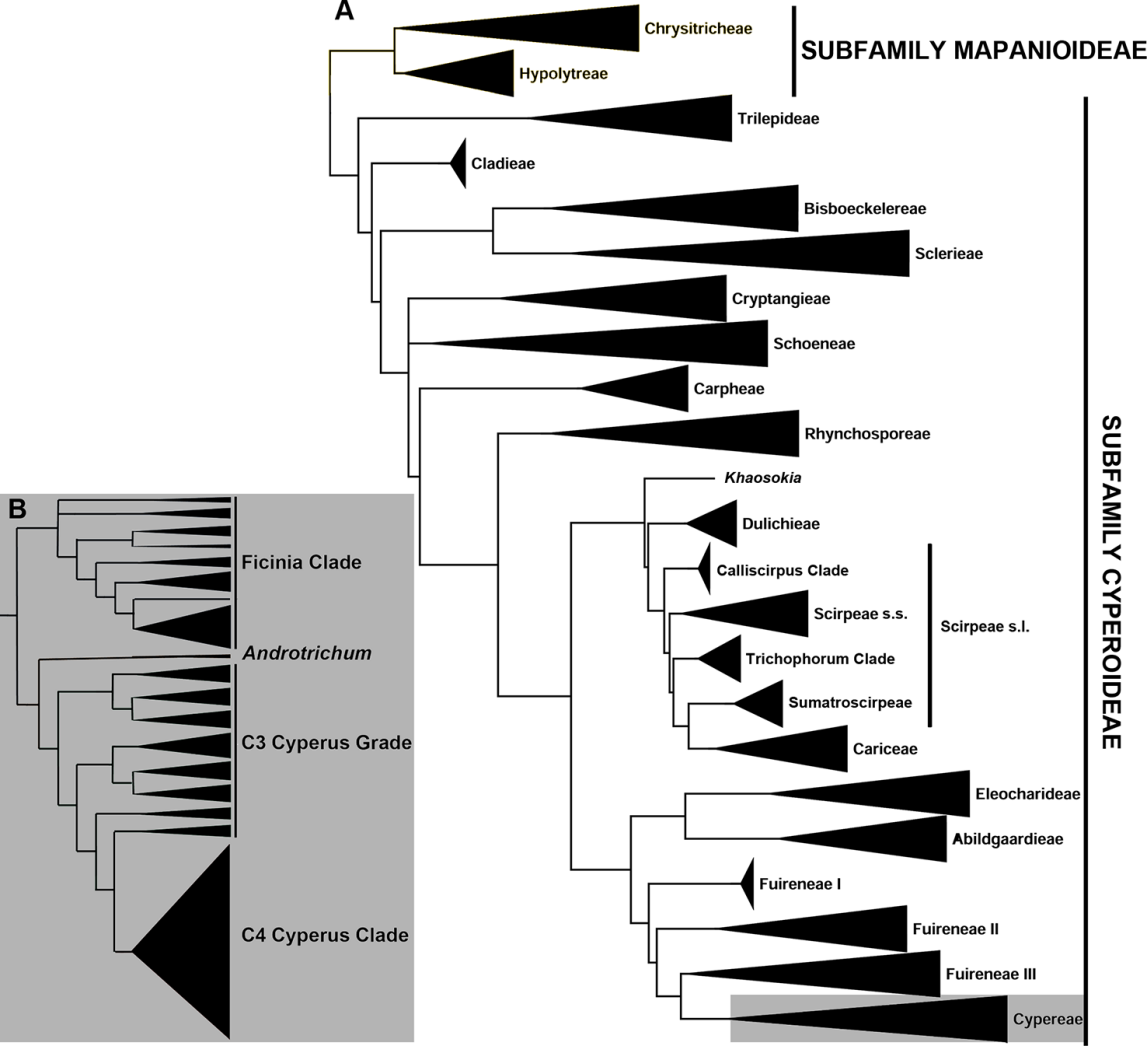
Phylogenetic relationships in Cyperaceae **(A)** Summary phylogeny of the family Cyperaceae based on Semmouri et al. (2019). **(B)** Summary phylogeny of the tribe *Cypereae* based on Larridon et al. (2011a, 2013).

Evolutionary relationships in the sedge family (Cyperaceae) have mainly been studied using Sanger sequencing (e.g., Simpson et al., 2007; Muasya et al., 2009; Jung and Choi, 2012; Escudero and Hipp, 2013; Spalink et al., 2016; Semmouri et al., 2019; **Figure 1A**). To date, high-throughput sequencing approaches in sedges include a targeted sequencing study using anchored hybrid enrichment or anchored phylogenomics (Lemmon et al., 2012; Buddenhagen et al., 2016), focusing on the Scirpo-Caricoid clade (Léveillé-Bourret et al., 2018), and two other studies using reduced-representation phylogenomic methods, restriction-site associated DNA sequencing (RAD-Seq; Baird et al., 2008), and genotyping-by-sequencing (GBS; Elshire et al., 2011; Deschamps et al., 2012), to resolve fine-scale relationships in the megadiverse genus *Carex* (Escudero et al., 2014; Maguilla et al., 2017). Hyb-Seq, i.e., targeted sequencing combined with genome skimming (Weitemier et al., 2014; Dodsworth et al., 2019), was recently used to investigate the broad-scale relationships in *Carex* (Villaverde et al., in review). Additionally, relationships at tribal and generic levels in Cyperaceae are being investigated using targeted sequencing (I. Larridon et al., unpubl. data). Relationships among Cyperaceae taxa that use the C4 photosynthetic pathway remain ill-understood, hampered by an apparent faster rate of diversification leading to limited topological resolution (e.g., Larridon et al., 2013; Bauters et al., 2014; Roalson et al., 2019). In particular, the relationships between C4 *Cyperus* L. species are still unresolved.

Within tribe *Cypereae*, the *Cyperus* clade includes two genera, i.e., the giant genus *Cyperus* (950 species) and the small genus *Androtrichum* (Brongn.) Brongn. (two species). Thirteen segregate genera recognized by Goetghebeur (1998) have since been subsumed into *Cyperus* (Larridon et al., 2011a; Larridon et al., 2011b; Larridon et al., 2013; Larridon et al., 2014; Bauters et al., 2014), comprising three genera that use C3 photosynthesis—*Courtoisina* Soják, *Kyllingiella* R.W. Haines & Lye, and *Oxycardium* Nees—and 10 genera that use C4 photosynthesis—*Alinula* J. Raynal, *Ascolepis* Nees ex Steud., *Ascopholis* C.E.C. Fisch., *Kyllinga* Rottb., *Lipocarpha* R. Br., *Pycreus* P. Beauv., *Queenslandiella* Domin, *Remirea* Aubl., *Sphaerocyperus* Lye, and *Volkiella* Merxm. & Czech. The small genus *Androtrichum* (C3 photosynthesis) has not yet been subsumed into *Cyperus* because only *rbcL* sequences are available to date, and the phylogenetic placement of the genus is consequently unresolved (Muasya et al., 2009; Jung and Choi, 2012; Hinchliff and Roalson, 2013; Semmouri et al., 2019). Given the ecological importance of *Cyperus*, which achieves high diversity and biomass in ecoregions across the tropics (Larridon et al., 2013; Kipkemboi and van Dam, 2018), and its ethnobotanical significance (Simpson and Inglis., 2001), understanding the diversity of the clade is of high societal importance.

Previous studies based on a limited set of plastid and nuclear ribosomal DNA (nrDNA) markers resolved relationships among species of *Cyperus* using the C3 photosynthetic pathway (Larridon et al., 2011a; Larridon et al., 2011b), but not among sections and species using C4 photosynthesis (Larridon et al., 2013; Larridon et al., 2014; Bauters et al., 2014). In these studies, the species-poor lineages of genus *Cyperus*, which diverge from deeper nodes, form a grade of generally well-circumscribed *Cyperus* sections that all use C3 photosynthesis (hereafter C3 *Cyperus* grade, c. 190 species); while the more highly derived clade (hereafter C4 *Cyperus* clade) represents a radiation of c. 760 *Cyperus* species that use C4 photosynthesis (**Figure 1B**). The C4 *Cyperus* clade is a particularly challenging lineage taxonomically (Huygh et al., 2010; Larridon et al., 2011c; Reynders et al., 2011; Bauters et al., 2014) and previous attempts to resolve relationships within it have resulted in a polytomy (Muasya et al., 2002; Larridon et al., 2011a; Larridon et al., 2013; Bauters et al., 2014). Spalink et al. (2016) showed an increased diversification rate for the C4 *Cyperus* clade commencing c. 20 Ma (million years ago).

It is still unclear under what conditions universal targeted sequencing kits, which target low-copy nuclear markers conserved across a wide phylogenetic range (e.g., angiosperms; Buddenhagen et al., 2016), can be used to infer relationships in fast evolving lineages. If they can, then there may be little benefit to developing custom probes for studies of small numbers of taxa, and there are obvious downstream benefits in using universal probes, data from which can be readily combined across labs. A recent study (Kadlec et al., 2017) on the heather genus *Erica* (Ericaceae, with well over 800 species) concluded that data from markers that are custom-designed using existing pipelines (e.g., MarkerMiner; Chamala et al., 2015) may deliver better results than those obtained using a more universal approach. As Liu et al. (2019) have shown, capture success drops significantly when probe sequences used in a targeted sequencing kit diverge >30% from their intended targets (**Supplementary Figures 5** and **6** in Liu et al., 2019). Recently, an angiosperm-wide targeted sequencing kit, i.e., Angiosperms-353 (Johnson et al., 2018), has been designed using a k-medoids clustering algorithm (Bauckhage, 2015) from a much larger dataset, including several published genomes (available from https://phytozome.jgi.doe.gov) plus 655 angiosperm transcriptomes generated by the one thousand plant transcriptomes (1KP) initiative (Matasci et al., 2014), in the context of the Plant and Fungal Trees of Life (PAFTOL) research program at Royal Botanic Gardens, Kew (https://www.kew.org/science/who-we-are-and-what-we-do/strategic-outputs-2020/plant-and-fungal-trees-life). The Angiosperms-353 kit (Johnson et al., 2018) targets 353 putatively single-copy protein-coding genes (spanning 260,802 bp in total) and was designed using transcriptome data from representatives of all major clades in angiosperms (three accessions belong in Cyperaceae—*Cyperus*, *Lepidosperma*, and *Mapania*—out of 128 monocots in total), to keep expected divergence between all potential taxa and the probes below the 30% divergence threshold beyond which capture is no longer efficient, as Liu et al. (2019) experimentally determined. This kit includes multiple probes for each locus (3x tiling) to optimize its performance with low-quality template (e.g., historical herbarium collections; Brewer et al., 2019). The aforementioned reasons may result in the Angiosperms-353 kit being more successful than other universal targeted sequencing kits for flowering plants.

Here, we present novel data from the C4 *Cyperus* clade obtained using both the Angiosperms-353 targeted sequencing kit and a Cyperaceae-specific kit designed by Villaverde et al. (in review) using the MarkerMiner pipeline (Chamala et al., 2015), with transcriptome data for *Cyperus papyrus* L. (1KP) and *Carex siderosticta* Hance (S. Kim et al., unpubl. data). The Cyperaceae-specific kit targets 554 low-copy nuclear orthologous loci, spanning c. 1 Mbp. We use these data to: 1) test the effectiveness of the Angiosperms-353 kit to resolve hitherto intractable relationships, 2) compare the relative effectiveness of these universal probes to the Cyperaceae-specific probes, and 3) establish well-supported relationships among this ecologically important group of sedges.

## Materials and Methods

### Taxon Sampling

Sampling for enrichment with the Angiosperms-353 kit consisted of 38 *Cyperus* accessions (one C3 *Cyperus* species, i.e., *Cyperus kyllingiella* Larridon, and 37 species from the C4 *Cyperus* clade) (**Supplementary Table 1A**). Sampling for enrichment with the Cyperaceae-specific kit consisted of eight species of the C4 *Cyperus* clade and *Schoenoplectus pungens* (Vahl) Palla (tribe Fuireneae) used as outgroup (**Supplementary Table 1B**). *Cyperus esculentus* L., *Cyperus mindorensis* (Steud.) Huygh, and *Cyperus richardii* Steud. were enriched with both kits.

### Deoxyribonucleic Acid Extraction, Library Preparation, Hybridization, and Sequencing

The voucher information and treatment of each accession is provided (**Supplementary Tables 1** and **2**). Both the Angiosperms-353 (Johnson et al., 2018) and the Cyperaceae-specific (Villaverde et al. in review) kits are available from Arbor Biosciences (Ann Arbor, MI, USA).

Molecular work for accessions enriched with the Angiosperms-353 probes was carried out at the Sackler Phylogenomic Laboratory, within the Jodrell Laboratory at Royal Botanic Gardens, Kew (Richmond, Surrey, UK). Genomic DNA was extracted from leaf tissue obtained from herbarium specimens or silica collected samples, using either a modified cetyl trimethylammonium bromide (CTAB) approach (Doyle and Doyle, 1987) or a CTAB protocol, based on Beck et al. (2012), modified for optimal simultaneous extraction of 96 to 192 samples (i.e., one or two plates) from suboptimal (i.e., herbarium) tissue (Fairlie & Pokorny protocol provided in **Supplementary Data Sheet 1**). Lastly, two accessions were sourced from the Kew DNA Bank (http://dnabank.science.kew.org/) (**Supplementary Table 1A**). The samples extracted using a CTAB approach were purified using Agencourt AMPure XP Bead Clean-up (Beckman Coulter, Indianapolis, IN, USA). All DNA extracts were quantified using a Quantus™ Fluorometer (Promega Corporation, Madison, WI, USA) and then run on a 1% agarose gel to assess the average fragment size. Samples with very low concentration (not visible on a 1% agarose gel), were assessed on an Agilent Technologies 4200 TapeStation System using Genomic DNA ScreenTape (Santa Clara, CA, USA). DNA extracts with average fragment sizes above 350 bp were sonicated using a Covaris M220 Focused-ultrasonicator™ (Covaris, Woburn, MA, USA) following the manufacturer’s protocol to obtain an average fragment size of 350 bp. Dual-indexed libraries for Illumina^®^ sequencing were prepared using the DNA NEBNext^®^ Ultra™ II Library Prep Kit and the NEBNext^®^ Multiplex Oligos for Illumina^®^ (Dual Index Primers Set 1 and 2) from New England BioLabs^®^ (Ipswich, MA, USA) following the manufacturer’s instructions but at half the recommended volumes. The quality of the libraries was evaluated on the TapeStation using High Sensitivity D1000 ScreenTape and the libraries were quantified using a Quantus Fluorometer. The final average library size including the adapters was c. 500 bp. Afterwards, the samples were pooled (8–24 samples/reaction) and enriched with the Angiosperms-353 probes (Johnson et al., 2018) following the manufacturer’s instructions (myProbes^®^ Manual v4.01, Arbor Biosciences, Ann Arbor, MI, USA) setting the hybridization temperature to 65°C for 24 h. Final products were again run on the TapeStation to assess quality (i.e., average fragment size) so they could be pooled equimolarly for sequencing (48–96 samples/pool). After multiplexing library pools, sequencing was performed on an Illumina^®^ MiSeq instrument (San Diego, CA, USA)—with v2 (300-cycles at 2 × 150 bp) or v3 (600-cycles at 2 × 300 bp) chemistry at Royal Botanic Gardens, Kew (Richmond, Surrey, UK)—or on an Illumina^®^ HiSeq (San Diego, CA, USA)—at either Macrogen (Seoul, South Korea) or GENEWIZ^®^ (Leipzig, Germany), producing 2 × 150 bp long reads.

Molecular work for the accessions enriched with the Cyperaceae-specific probes was carried out at The Morton Arboretum (Lisle, IL, USA) and the Pritzker Laboratory of the Field Museum of Natural History (Chicago, IL, USA). Genomic DNA was extracted from leaf tissue obtained from silica preserved samples (**Supplementary Table 1B**) using the QIAGEN DNeasy Plant Mini Kit following the manufacturer’s protocols (QIAGEN, Valencia, CA, USA) or a modified CTAB protocol (Doyle and Doyle, 1987). Samples were sonicated to a target fragment size of 550 bp using a Covaris E220 Focused-ultrasonicator™ (Wohurn, MA, USA). Sequencing libraries were prepared using the Illumina^®^ TruSeq Nano HT DNA kit (San Diego, CA, USA). DNA libraries were checked for quality using an Agilent Technologies 2100 Bioanalyzer (Santa Clara, CA, USA) and their concentration quantified using a Qubit 2.0 Fluorometer (Life Technologies, Grand Island, NY, USA). Indexed samples were pooled in approximately equal quantities and the pool was enriched using the custom Cyperaceae-specific probes (Villaverde et al. in review) following the manufacturer’s protocols for myBaits^®^ kit (v3), i.e., we hybridized at 65°C for 16 h. The paired-end libraries were sequenced in one run (including a total of 88 accessions; Villaverde et al. in review) on an Illumina MiSeq (2 × 300 bp; 600 cycle v3) at the Pritzker Laboratory.

### Bioinformatics, Contig Assembly, and Multi-Sequence Alignment

Raw reads were trimmed to remove adapter sequences and to remove portions of low quality with Trimmomatic v.0.36 (Bolger et al., 2014) using the setting LEADING:20 TRAILING:20 SLIDINGWINDOW:4:20 MINLEN:50. HybPiper v1.3.1 (Johnson et al., 2016) was used with default settings to process the quality-checked, trimmed reads. Paired reads of samples enriched with two different kits independently (Angiosperms-353 and Cyperaceae-specific) were mapped to targets using BWA v0.7.17 (Li and Durbin, 2009) and their respective nucleotide (DNA) target file (**Supplementary Data Sheet 2**); additionally, we also used BLASTx (Altschul et al., 1990) when using the Angiosperms-353 target loci with amino acid (AA) sequences (**Supplementary Data Sheet 3**). Summary statistics such as the percentage of reads mapping were generated using SAMtools flagstat v1.8 (Li et al., 2009). Mapped reads were then assembled into contigs with SPAdes v3.13.1 (Bankevich et al., 2012). Subsequently, exonerate v2.2 (Slater and Birney, 2005) was used to align the assembled contigs to their associated target sequence and remove intronic regions. Only exon data were analyzed in the current study in order to directly compare the information content provided by the targeted loci. HybPiper flags potential paralogs when multiple contigs are discovered mapping well to a single reference sequence. The program uses coverage and identity to a reference to choose a “main” sequence and denotes the others as potential paralogs. All loci flagged as potential paralogs were removed from downstream analyses. A list of the potential paralogs is provided (**Supplementary Table 3**).

The paralog-filtered consensus sequences for each locus were used to generate eight different datasets (**Table 1**). This allowed us to investigate the phylogenetic informativeness of the data generated by the two kits separately and allowed us to test the mergeability of the data generated by both kits. Four unmerged datasets were created: (dataset 1) loci targeted with the Angiosperms-353 kit for the 37 C4 *Cyperus* clade accessions enriched with this kit plus *Cyperus kyllingiella* (C3 *Cyperus* grade) as outgroup; (dataset 2) loci targeted with the Cyperaceae-specific baits for the eight C4 *Cyperus* clade accessions enriched with this kit plus *S. pungens* as outgroup; (dataset 3) loci targeted with the Angiosperms-353 kit for a subset of eight C4 *Cyperus* clade accessions enriched with this kit; (dataset 4) loci targeted with the Cyperaceae-specific baits for the eight C4 *Cyperus* clade accessions enriched with this kit. Four merged datasets were assembled: (dataset 5) loci targeted with the Angiosperms-353 baits for all 46 *Cyperus* accessions; (dataset 6) loci targeted with the Cyperaceae-specific kit for all 46 *Cyperus* accessions; (dataset 7) all targeted loci for all 46 *Cyperus* accessions; and (dataset 8) the 57 overlapping loci targeted by both bait kits for all 46 *Cyperus* accessions (retrieved from the Angiosperms-353 data). The overlapping loci are listed in **Supplementary Table 4**. Contigs were aligned using MAFFT v7 (Katoh and Standley, 2013) with the “–auto” option. The number of parsimony informative sites were calculated for each contig alignment using AMAS (Borowiec, 2016).

**Table 1:** The eight datasets analyzed in this study.

	Loci targeted by	Accessions enriched with	Accessions included
Unmerged datasets	Angiosperms-353	Angiosperms-353	37 C4 *Cyperus* clade + *Cyperus kyllingiella* as outgroup
	Cyperaceae-specific	Cyperaceae-specific	8 C4 *Cyperus* clade + *Schoenoplectus pungens* as outgroup
	Angiosperms-353	Angiosperms-353	8 C4 *Cyperus* clade
	Cyperaceae-specific	Cyperaceae-specific	8 C4 *Cyperus* clade
Merged datasets	Angiosperms-353	All accessions	46 *Cyperus* accessions
	Cyperaceae-specific	All accessions	46 *Cyperus* accessions
	All loci	All accessions	46 *Cyperus* accessions
	57 overlapping loci	All accessions	46 *Cyperus* accessions

Dataset 3 and dataset 4 were analyzed to account for the difference in sampling size when comparing the number of PIS retrieved across locus alignments for the two targeted sequencing kits. In these datasets, the eight C4 *Cyperus* clade accessions from dataset 2 (representing loci targeted with the Cyperaceae-specific baits for the accessions enriched with this kit) were compared with a taxonomically equivalent subset of eight accessions from dataset 1 (representing loci targeted with the Angiosperms-353 kit for the accessions enriched with this kit). The accessions selected for this subsampling are indicated by an asterisk in **Supplementary Tables 1** and **2**, and are represented respectively in dataset 3 and dataset 4 by 1) four species of the former segregate genus *Kyllinga* plus a closely related species to match five species of former segregate genus *Kyllinga* (two of which are represented by the same species in both datasets); 2) one species of the former segregate genus *Ascolepis* to match one species of the former segregate genus *Ascolepis*; 3) *C. esculentus* (represented by the same accession in both datasets); and 4) *C. papyrus* L. to match *Cyperus rotundus* L. which are closely related species.

### Phylogenetic Inference

Trees were inferred using either a summary method that is statistically consistent under the Multiple Species Coalescent (MSC) (i.e., ASTRAL-III) or a total evidence approach, in which maximum likelihood (ML) inference was conducted on a concatenated matrix of all loci. Both approaches were used to analyze the eight different datasets described above (**Table 1**). For the summary approach under the MSC, individual gene trees were constructed using RAxML v8 (Stamatakis, 2014) applying GTRCAT and 200 bootstrap replicates followed by slow ML optimization with the “-f a” option. We then ran ASTRAL-III v5.5.11 (Zhang et al., 2018) to infer a species tree using “-t 2” to output quartet support values visualizing gene tree conflict. For the total evidence approach, phylogenetic inference of the targeted sequencing data was executed in IQ-TREE v1.6.10 (Nguyen et al., 2015) with 1,000 ultrafast bootstraps using the “- bb” and “-m TEST” options. To investigate gene tree *versus* species tree concordance, we calculated quartet distance between each individual gene tree and the concatenated total evidence tree obtained using the R package Quartet v1.0.2 (Sand et al., 2014; Smith, 2019), which yields a measure of the similarity of each gene tree *versus* the species tree based on shared four-taxon subtrees. We also calculated two measures of genealogical concordance in our dataset, the gene concordance factor (gCF) and the site concordance factor (sCF), using the options “-gcf” and “-scf” in IQ-TREE v1.7beta (Nguyen et al., 2015; Minh et al., 2018). This approach provides a description of possible disagreement among loci and across sites.

## Results

### Targeted Sequencing Kits and Data Quality

Summary statistics are available in **Supplementary Table 5**. When comparing the summary statistics between the two equally sized datasets 3 and 4 (**Supplementary Table 5C**), on average 324,655 (42,386–832,980) paired reads were produced for the accessions enriched with Angiosperms-353 probes *vs.* 199,961 (52,978–420,228) for the accessions enriched with the Cyperaceae-specific probes. Raw reads for all accessions are available from GenBank Sequence Read Archive (SRA) under BioProject numbers PRJNA553989 (*Cyperus* BioProject) and PRJNA553631 (*S. pungens*—*Carex* BioProject) (http://www.ncbi.nlm.nih.gov/bioproject/PRJNA553989; http://www.ncbi.nlm.nih.gov/bioproject/PRJNA553631).

### Universal *Versus* Family-Specific Probes

**Figure 2:**
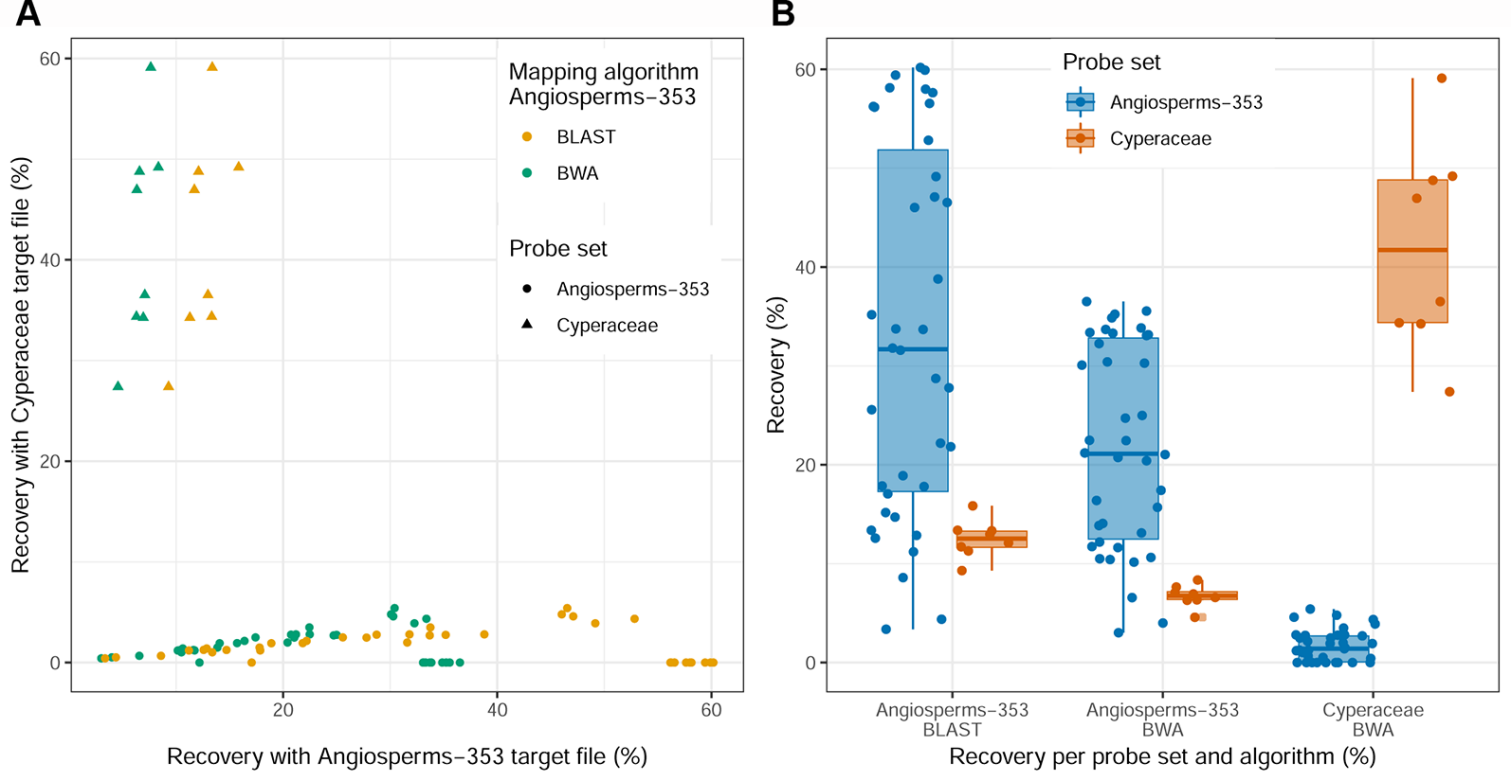
Recovery success for the Angiosperms-353 kit *vs.* the Cyperaceae-specific kit. **(A)** Percentage recovery with Angiosperms-353 (AA) target file from the accessions enriched with the Angiosperms-353 kit *vs.* percentage recovery of the Cyperaceae-specific targets from the accessions enriched with the Cyperaceae-specific probes. **(B)** Summary of recovery success per kit and across kits.

**Figure 3:**
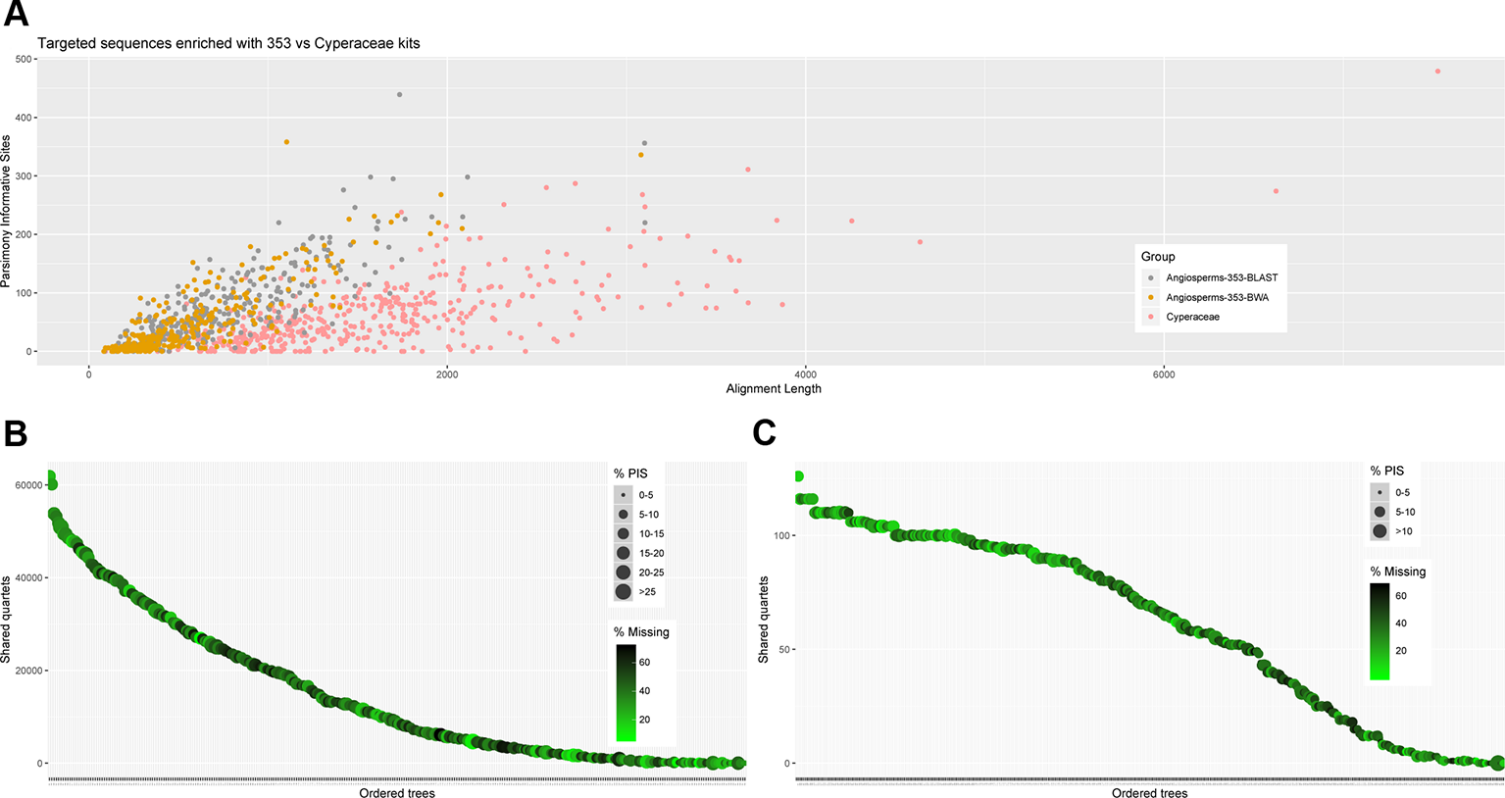
**(A)** Scatter plot of aligned contig length *versus* number of parsimony informative sites. **(B)** Number of gene trees that support the inferred species tree for data generated with the Angiosperms-353 probes, and **(C)** for data generated with the Cyperaceae-specific kit.

Recovery success and sequence length of the targeted loci, with both targeted sequencing kits, are provided in **Supplementary Table 2** and visualized in **Figure 2** and **Supplementary Images 1**–**3**. Percentage of recovery, i.e., the percentage of summed captured length of all target loci per individual divided by the summed mean length of all loci, was highest when running HybPiper for accessions enriched with the Cyperaceae-specific kit with its corresponding nucleotide target file (42.1%). We recovered on average 396 loci (324–476; **Supplementary Table 2C**) with the Cyperaceae-specific kit. For accessions enriched with the Angiosperms-353 kit, capture success was higher with the AA target file (33.23%) than with the DNA target file (21.7%). We recovered on average 215 loci (39–309; **Supplementary Table 2B**) with the AA target file and 162 loci (35–235) with the DNA target file. For data generated with the Angiosperms-353 kit, post-alignment length of contigs ranged from 87 to 3,103 bp long, with 751 bp mean length (**Table 2**; **Supplementary Table 6A**). For data generated with the Cyperaceae-specific kit, post-alignment length of contigs ranged from 93 to 7,527 bp long, with 1,608 bp mean length (**Table 2**; **Supplementary Table 6B**). In both cases, longer contigs had more Parsimony Informative Sites (PIS) (**Figure 3**).

**Table 2:** Length of the aligned contigs and number of parsimony informative sites (PIS) for data obtained after enrichment with the Angiosperms-353 and Cyperaceae-specific probes.

		Contig	PIS
Angiosperms-353	Mean	751	75
(38 accessions)	SD	438	65
*Dataset 1*	Min	87	0
	Max	3,103	439
	**Total**	**233,429**	**23,217**
Cyperaceae-specific	Mean	1,608	63
(9 accessions)	SD	830	59
*Dataset 2*	Min	93	0
	Max	7,527	479
	**Total**	**683,427**	**26,630**
Angiosperms-353	Mean	717	25
(subset of 8	SD	411	28
accessions)	Min	150	0
*Dataset 3*	Max	2,826	147
	**Total**	**221,564**	**7,613**
Cyperaceae-specific	Mean	1,471	50
(8 accessions)	SD	818	51
*Dataset 4*	Min	162	0
	Max	7,524	400
	**Total**	**667,945**	**22,767**

**Figure 4:**
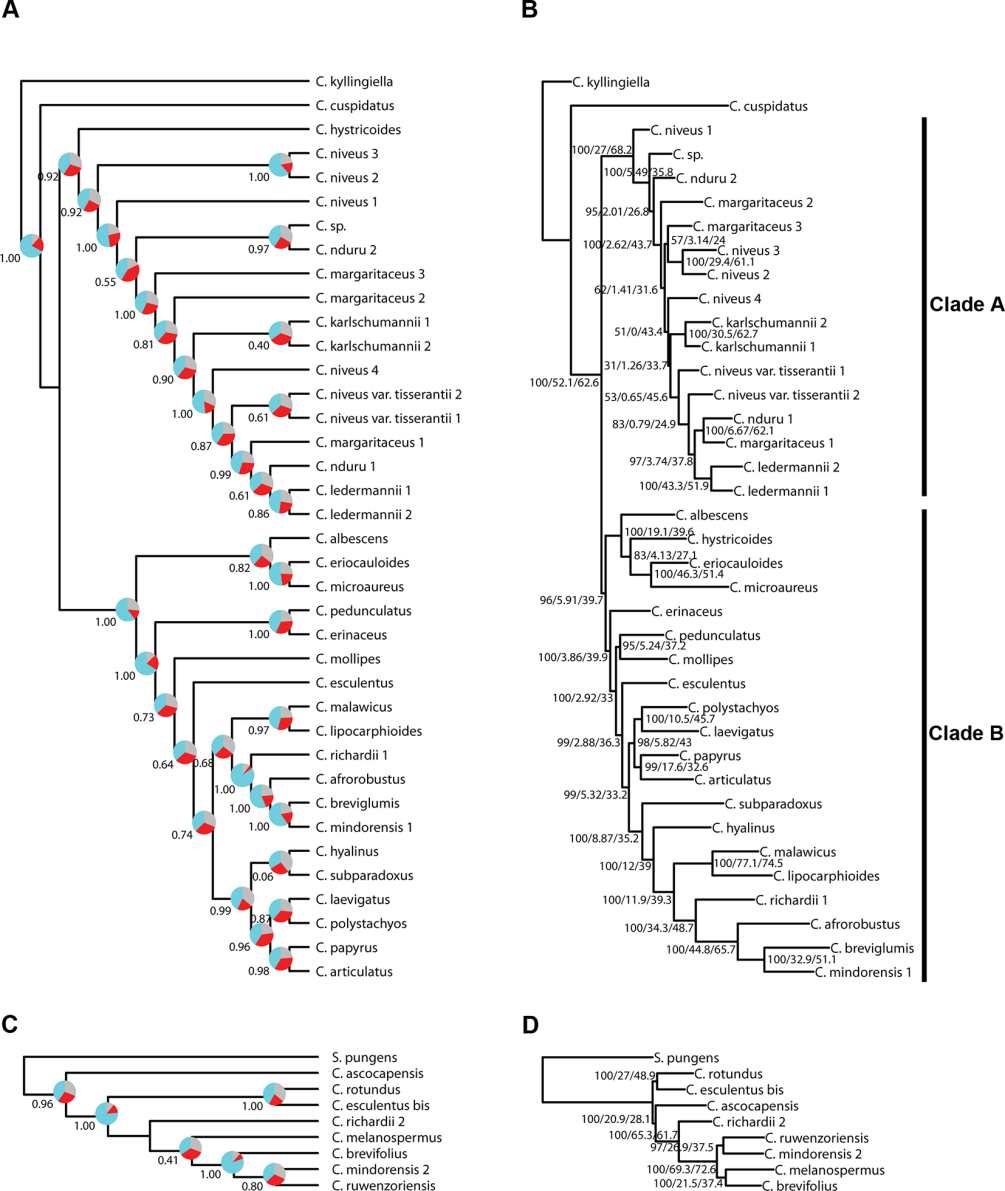
Phylogenetic reconstructions using ASTRAL **(A, C)** and using IQ-TREE **(B, D)** of the relationships in the C4 *Cyperus* clade inferred for **(A, B)** dataset 1, i.e., 38 samples enriched with the Angiosperms-353 probes, and **(C, D)** dataset 2, i.e., 9 samples enriched with the Cyperaceae-specific probes. The ASTRAL trees show local posterior probability values and pie charts visualizing quartet support values at the nodes (blue = agreeing loci; red = disagreeing loci; gray = uninformative), while the IQ-TREE trees show BS/gCF/sCF values at the nodes.

We investigated three measures of resolution power when dealing with shallow-level phylogenetic relationships for the Angiosperms-353 kit *versus* the Cyperaceae-specific kit: 1) the proportion of gene trees that support the inferred species tree (under the MSC) (**Figure 3**; **Figures 4A, C**), 2) the disagreement among loci and across sites in the total evidence tree (**Figures 4B, D**), and 3) the number of PIS retrieved across locus alignments (**Figure 3**; **Supplementary Images 4** and **5**). Addressing the first measure, many nodes are well supported in the ASTRAL tree generated for dataset 1 (loci targeted with the Angiosperms-353 kit for the accessions enriched with this kit) (**Figure 4A**) having local posterior probability (LPP) values greater than 0.9. Addressing the second measure, many nodes are similarly well supported in the IQ-TREE tree generated with the same data (**Figure 4B**) with most nodes having bootstrap (BS) values greater than 90%. However, some of the branches that received low LPP or BS value support have quartet scores indicating gene tree conflict and/or have low gCF scores, which indicates that few gene trees support the grouping. These branches occur among some species of the *Cyperus margaritaceus*-*Cyperus niveus* complex (clade A) and in the main backbone of clade B, which represents species of C4 *Cyperus* s.s. and the 10 C4 segregate genera accepted by Goetghebeur (1998). In the phylogenetic hypotheses obtained for dataset 2 (loci targeted with the Cyperaceae-specific probes for the accessions enriched with this kit) (**Figures 4C, D**), most nodes are equally well supported having LPP values greater than 0.9 or BS values greater than 90%. Addressing the third measure, when comparing the probe sets in terms of PIS by comparing dataset 4 (loci targeted by the Cyperaceae-specific probes for the eight C4 *Cyperus* clade accessions enriched with this kit; **Supplementary Table 6D**) with the equally sized and taxonomically equivalent dataset 3 (loci targeted by the Angiosperms-353 probes for a subset of eight C4 *Cyperus* clade accessions enriched with this kit; **Supplementary Table 6C**), the former has an average contig length of 1,471 (162–7,524), while the latter has shorter average length of 717 (150–2,826). However, the relative number of PIS is the same at 0.03 PIS/bp. This is also shown in **Supplementary Image 4**. A comparison of the support provided by the gene trees for the species tree between the two datasets of eight accessions is provided in **Supplementary Image 5**.

### Mergeability of Data Obtained With Different Targeted Sequencing Kits

Different percentages of recovery were obtained using the AA or the DNA target files of the Angiosperms-353 kit in the accessions enriched with the Cyperaceae-specific kit. With the AA target file, we were able to recover 12.5% of the total target size from accessions enriched with the Cyperaceae-specific kit (**Supplementary Table 2B**). This percentage decreases to 6.73% when using the DNA target file (**Supplementary Table 2A**). On average 44 loci (35–53) were retrieved from accessions enriched with the Cyperaceae-specific kit using the Angiosperms-353 AA target file, and 32 (21–41) with the DNA target file. Capture success was very low when targeting Cyperaceae-specific loci from accessions enriched with the Angiosperms-353 probes (1.7%) using the DNA target file; however, sequence data was still retrieved from an average of 37 loci (0–106; **Supplementary Table 2C**). This information is summarized in **Figure 2**.

We tested the mergeability of the data generated for all *Cyperus* samples produced after enrichment with the two targeted sequencing kits, by inferring trees using ASTRAL and IQ-TREE for four merged datasets. **Table 3** provides the length of the aligned contigs and number of PIS for the four merged datasets. The number of PIS is positively correlated with the size of the dataset (**Table 3**; **Supplementary Tables 6E–H**).

**Table 3:** Length of the aligned contigs and number of parsimony informative sites (PIS) for the four merged datasets: 1) aligned contigs of the loci targeted with the Angiosperm-353 probes, 2) aligned contigs of the loci targeted with the Cyperaceae-specific probes, 3) aligned contigs of all loci (without duplicating the overlapping genes), and 4) aligned contigs of the 57 overlapping loci targeted by both kits.

		Contig	PIS
Angiosperms-353	Mean	739	85
*Dataset 5*	SD	492	75
	Min	87	0
	Max	4,211	539
	**Total**	**259,321**	**27,656**
Cyperaceae-specific	Mean	1,458	76
*Dataset 6*	SD	805	81
	Min	153	0
	Max	7,520	554
	**Total**	**720,324**	**37,645**
All loci	Mean	1,192	74
*Dataset 7*	SD	775	74
	Min	87	0
	Max	7,520	554
	**Total**	**910,926**	**56,615**
57 overlapping loci	Mean	1,206	152
*Dataset 8*	SD	687	100
	Min	312	6
	Max	4,211	539
	**Total**	**68,719**	**8,686**

**Figure 5:**
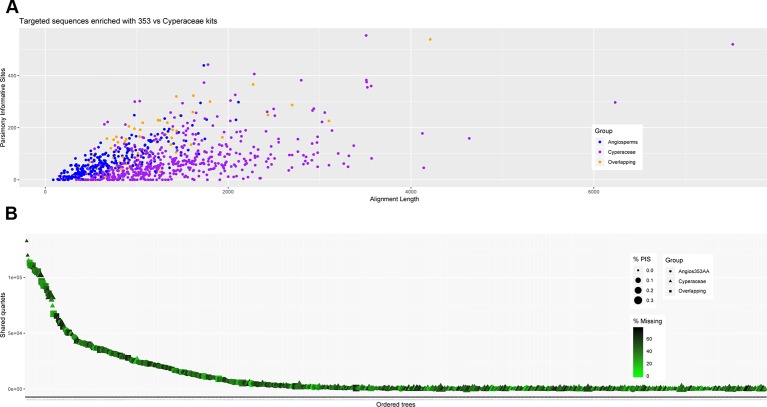
Number of gene trees that support the inferred species tree **(A)**; and **(B)** scatter plot of aligned contig length *vs.* parsimony informative sites (PIS) for the data generated for all samples recovered targeting the Angiosperms-353 genes, the Cyperaceae-specific genes, and indicating the overlapping genes.

The amount of data retrieved targeting the Cyperaceae-specific loci from the data generated for all samples is larger when comparing the length of the alignments (**Table 3**; **Figure 5**) and the total number of PIS is also higher (c. 26.5% more PIS). The 57 overlapping loci are on average longer than those of most loci targeted by the Angiosperms-353 probes (1,206 *versus* 793) but shorter than the average for loci targeted with the Cyperaceae-specific kit (1,206 *versus* 1,458). These overlapping loci have a higher proportion of PIS per alignment in comparison with the other merged datasets (152 *versus* 74–85) (**Supplementary Image 6**; **Supplementary Tables 6E–H**). Although it includes much less data than the other analyzed datasets, the dataset of the 57 overlapping loci still includes a high number of PIS (8,686 PIS out of 68,719 bp or 0.12 PIS/bp; **Table 3**; **Supplementary Table 6H**).

**Supplementary Table 4** lists the names of the overlapping loci for both targeted sequencing kits and a comparison of statistics between the recovery of these loci is provided in **Supplementary Table 6I**. The average contig length of the overlapping genes retrieved with the Angiosperms-353 AA target file is shorter than when they are retrieved with the Cyperaceae-specific DNA target file (1,206 *vs.* 1,482). However, the average number of PIS retrieved with the Angiosperms-353 AA target file is higher than when the data are retrieved with the Cyperaceae-specific DNA target file (152 *vs.* 126).

### Resolving Relationships in the C4 *Cyperus* Clade

Topologies produced with both approaches for dataset 1 (loci targeted with the Angiosperms-353 kit for the accessions enriched with this kit; **Figures 4A, B**) are very similar, except for the position of *Cyperus hystricoides* (B. Nord.) Bauters, which is retrieved as sister to clade A in the ASTRAL analysis (**Figure 4A**) and as part of clade B in the IQ-TREE analysis (**Figure 4B**). Likewise, the trees generated with both approaches, using dataset 2 (loci targeted with the Cyperaceae-specific probes for the accessions enriched with this kit; **Figures 4C, D**), result in similar topologies, except for the placement of *Cyperus ascocapensis* Bauters.

**Figure 6:**
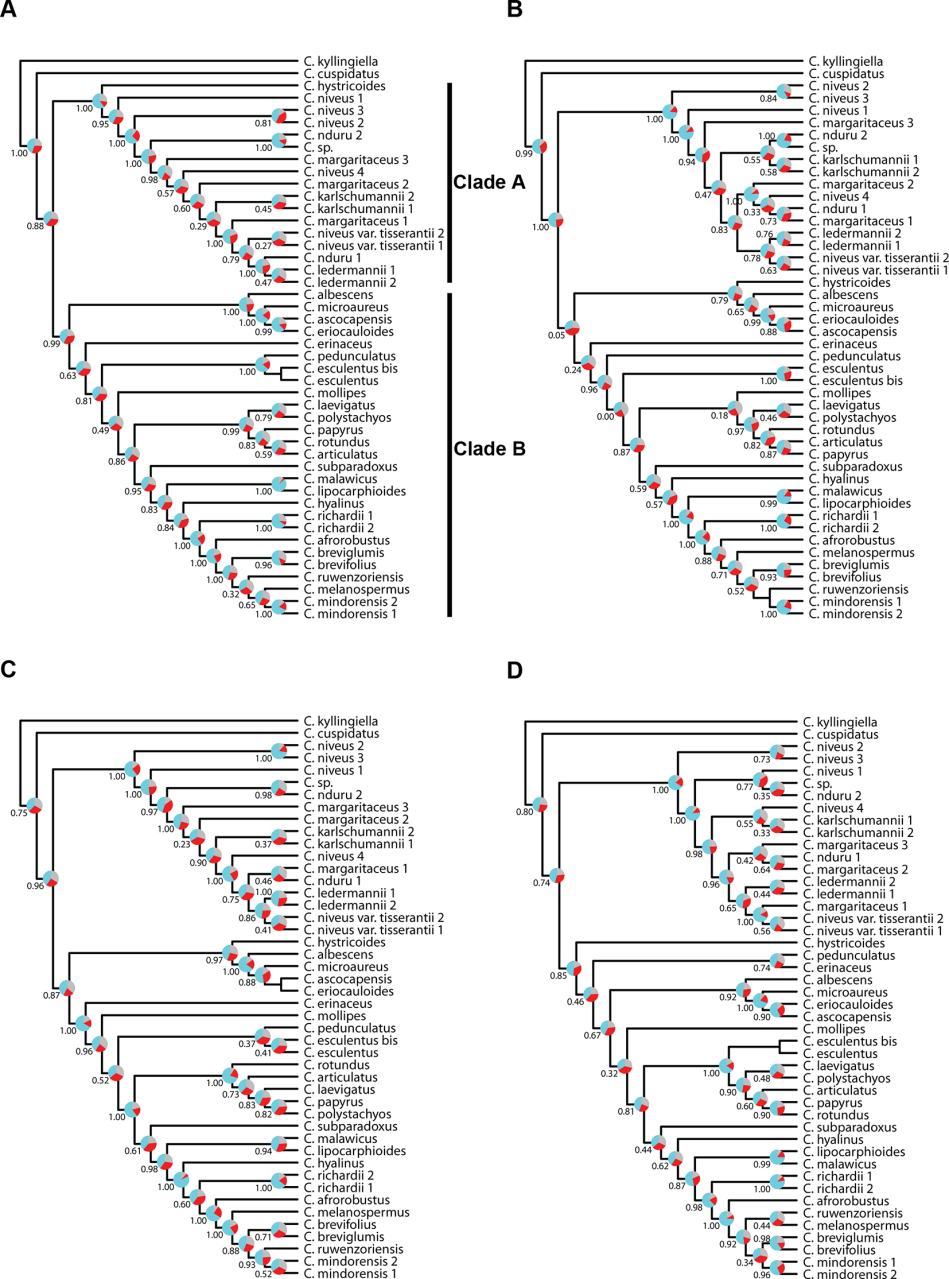
Phylogenetic reconstructions using ASTRAL of the relationships in the C4 *Cyperus* clade inferred for all accessions from aligned contigs of **(A)** dataset 5, i.e., the loci targeted with the Angiosperms-353 probes, **(B)** dataset 6, i.e., the loci targeted with the Cyperaceae-specific kit, **(C)** dataset 7, i.e., all targeted loci, and **(D)** dataset 8, i.e., the overlapping loci targeted by both kits. The trees show local posterior probability values and pie charts visualizing quartet support values at the nodes (blue = agreeing loci; red = disagreeing loci; gray = uninformative).

The topologies in the ASTRAL trees resulting from the four merged datasets are very similar (**Figure 6**), with generally high levels of node support. As in **Figure 4**, the placement of *C. hystricoides* was unstable, being reconstructed as sister to clade A in the tree based on the loci targeted by the Angiosperms-353 kit (**Figure 6A**), and among the first branching lineages of clade B in the other analyses (**Figures 6B, D**). Other differences in topology were found among taxa of the *C. margaritaceus*-*C. niveus* complex (clade A) and in the backbone of clade B where node support is lower and quartet scores indicate higher gene tree discordance (**Figure 6**). The proportion of gene trees supporting the retrieved topology is similar in **Figures 6A–C**, but the ASTRAL analysis of dataset 8 (overlapping loci for all accessions), yields a tree with higher locus concordance at most nodes.

**Figure 7:**
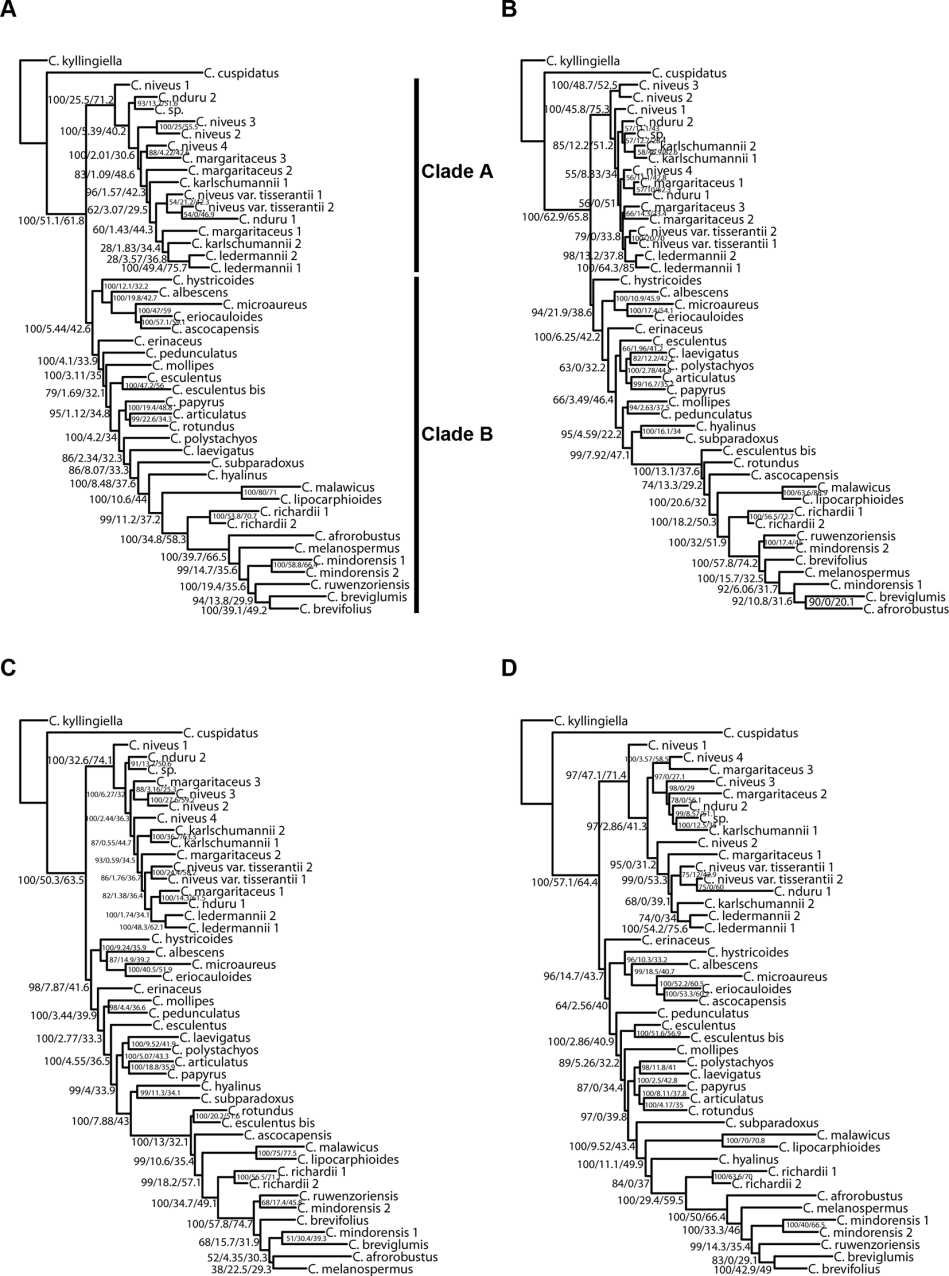
Phylogenetic reconstructions using IQ-TREE of the relationships in the C4 *Cyperus* clade inferred for all accessions from aligned contigs of **(A)** dataset 5, i.e., the loci targeted with the Angiosperms-353 probes, **(B)** dataset 6, i.e., the loci targeted with the Cyperaceae-specific kit, **(C)** dataset 7, i.e., all targeted loci, and **(D)** dataset 8, i.e., the overlapping loci targeted by both kits. The trees show bootstrap/gene concordance factor/site concordance factor values at the nodes.

The analyses performed with IQ-TREE yielded topologies similar (**Figure 7**) to those retrieved with ASTRAL for the respective datasets. For clade A, a morphologically homogeneous species complex in the C4 *Cyperus* clade, resolution, and support are comparable between the ASTRAL and IQ-TREE results (**Figures 6** and **7**). However, for clade B, a morphologically heterogeneous subset of the C4 *Cyperus* clade, the IQ-TREE analyses provided higher support for some nodes, although the IQ-TREE topology is often less well resolved (**Figures 6B, D** *vs.* **Figures 7B, D**). For dataset 6 (loci targeted with the Cyperaceae-specific probes for all accessions) and dataset 7 (all loci for all accessions), ASTRAL, a summary species-tree method, seems the handle high levels of missing data better in that the obtained results retrieve conspecific accessions or closely related species as sister taxa (**Figures 6B, C**). On the other hand, the total evidence trees inferred from concatenated data matrices in IQ-TREE under ML does not accurately place several samples in clade B (**Figures 7B, C**), i.e., *C. esculentus* (same accession sequenced with both targeted sequencing kits) is not reconstructed as monophyletic, and neither is *C. mindorensis* (different accessions but the same species). Similarly, the IQ-TREE analysis of dataset 8 (overlapping loci for all accessions; **Figure 7D**), did not reconstruct *Cyperus ledermannii* (Kük.) S.S. Hooper, *C. niveus* var. *tisserantii* (Cherm.) Lye, *C. mindorensis*, and *C. richardii* as monophyletic. This issue is not found for taxa of clade B in the IQ-TREE ML analysis of dataset 5 (loci targeted with the Angiosperms-353 kit for all accessions), although placement of *Cyperus karlschumannii* C.B. Clarke and *C. niveus* var. *tisserantii* were not reconstructed as expected in clade A (**Figure 7A**).

When considering gene tree concordance for the analyses of dataset 5 (loci targeted with the Angiosperms-353 kit for all accessions), the monophyly of conspecific accessions or accessions of closely related species [e.g., *Cyperus lipocarphioides* (Kük.) Lye and *Cyperus malawicus* (J. Raynal) Lye] in clade B is supported by a high number of gene trees (**Figure 7A**). In clade A, only a few taxa are resolved as monophyletic (**Figure 7A**). In contrast, in the IQ-TREE results of dataset 6 (loci targeted with the Cyperaceae-specific probes for all accessions), and in the analysis of dataset 7 (all loci for all accessions), conspecific accessions in clade A tend to be retrieved as monophyletic and supported by a significant number of gene trees, while phylogenetic relationships between the taxa of clade B are not well resolved (**Figures 7B, C**). In the analyses based on dataset 8 (overlapping loci for all accessions), most nodes in clade B are supported by a proportion of gene trees, while many nodes in clade A have a gCF value of 0 (**Figure 7D**). This result contrasts with the higher locus concordance observed at most nodes in the ASTRAL analysis of this dataset (**Figure 6D**).

## Discussion

### Data Quality: Herbarium *Versus* Tissue Preserved for Deoxyribonucleic Acid Extraction

Most tissue samples in this study (30 out of 47) were obtained from herbarium specimens. The remainder were from silica dried samples (17 samples). The high quality of the sequence capture across types illustrates the potential of targeted sequencing to generate genomic level data from fragmented DNA (Hart et al., 2016; McKain et al., 2018; Villaverde et al., 2018; Brewer et al., 2019). Differences in extraction and sequencing methods did not appear to influence capture success or recovered target length with respect to total length targeted (**Supplementary Table 2**).

### Universal *Versus* Family-Specific Probes

Recovery of loci targeted with the Cyperaceae-specific probes from accessions enriched with this kit was higher than for loci targeted with the universal Angiosperms-353 kit (**Supplementary Table 2**). Although more data was retrieved with the Cyperaceae-specific kit (dataset 2) than with the Angiosperms-353 kit (dataset 1) (total length aligned contigs was 683,427 bp and 233,429 bp, respectively), the number of PIS was comparable (26,630 *vs.* 23,217, respectively), yielding a lower ratio of PIS per total length aligned for the Cyperaceae-specific kit (0.04 PIS/bp) than for the Angiosperms-353 kit (0.1 PIS/bp) (**Table 2**). However, when comparing the values between the equally sized and taxonomically equivalent datasets 3 and 4, a comparable ratio of PIS per total aligned contig length is obtained (both with 0.03 PIS/bp). This indicates that the power of both targeted sequencing kits (off-target reads notwithstanding) to resolve shallow-level relationships for the C4 *Cyperus* clade is quite similar (**Figure 3**; **Table 2**).

Our finding belies our expectation going into this study that our family-specific kit would recover more variable loci. A recent study (Kadlec et al., 2017) on the species-rich heather genus *Erica* concluded that data from markers, custom-designed using the MarkerMiner pipeline, deliver better results than those obtained using a more universal approach. In another recent study, Chau et al. (2018) developed a custom-designed targeted sequencing kit using data from two *Buddleja* species (Scrophulariaceae) from 1KP using a modified version of the Sondovac marker development pipeline (Schmickl et al., 2016). They compared these taxon-specific loci with three universal loci sets (a conserved ortholog set [COSII] by Wu et al., 2006, shared single-copy nuclear [APVO SSC] genes by Duarte et al., 2010; and pentatricopeptide repeat [PPR] genes by Yuan et al., 2009). Chau et al. (2018) conclude that targeted sequencing is an effective method for increasing resolution and support in phylogenetics compared to Sanger sequencing, and that universal target loci can be as effective as taxon-specific loci in terms of capture success and phylogenetic informativeness. Our results support the conclusions of Chau et al. (2018). The advantage of a universal off-the-shelf targeted sequencing kit like the Angiosperms-353 kit is that it opens the opportunity to use targeted sequencing in plant groups with few genomic resources (Chau et al., 2018). Furthermore, universal kits are attractive in terms of reduced cost and effort, because they generate data suitable for wider analyses across angiosperms and may be applied as a DNA barcoding tool (Blattner, 2016; Kadlec et al., 2017), and predesigned kits can often be purchased at a discount from the producers (https://arborbiosci.com/products/mybaits-plant-angiosperms/).

### Mergeability of Data Obtained with Different Targeted Sequencing Kits

Villaverde et al. (in review) combined accessions enriched with the anchored phylogenomics probes (Buddenhagen et al., 2016; Léveillé-Bourret et al., 2018) in their analyses of *Carex* using the Cyperaceae-specific kit. Here, we go one step further: we combine accessions enriched with two different kits, and we merge the data in both directions. Our study may be the first to do so, at least in angiosperms. As expected, sequence data recovery is higher when analyses are performed with the target file that matches the loci for which the samples were enriched (**Figure 2**). Nonetheless, even with limited overlap (57 genes) between targeted sequencing kits and a high level of missing data, data analyses under the MSC recover the same overall topology irrespective of dataset, with strong support. However, combined data analyses using a concatenated ML approach appear less robust to dataset differences and result in conspecific accessions not always being reconstructed as monophyletic when analyzing the merged datasets.

### Resolving Relationships in the C4 *Cyperus* Clade

Most nodes are well supported in all analyses we conducted in the C4 *Cyperus* clade (**Figures 4**, **6**, and **7**), except for the branches near the backbone of clade B, as has been observed in earlier studies (e.g., Larridon et al., 2013; Bauters et al., 2014; Semmouri et al., 2019). These nodes show a higher gene tree discordance (based on ASTRAL quartet score and IQ-TREE gCF and sCF values), which likely resulted from an increased diversification rate (Spalink et al., 2016). Still the resolution and support retrieved in the backbone of the C4 *Cyperus* clade from targeted sequencing data is an important improvement over the polytomy obtained with Sanger sequencing results (e.g., Larridon et al., 2013).

The relationships retrieved in the C4 *Cyperus* clade, here investigated for the first time using phylogenomic data, largely match those obtained in previous studies (e.g., Larridon et al., 2011b; Larridon et al., 2013), with *Cyperus cuspidatus* Kunth sister to all other taxa in the C4 *Cyperus* clade. This species has an inflorescence of digitately clustered spikelets, which is characteristic of species in the C3 *Cyperus* grade and C4 *Cyperus* section *Amabilis* C.B. Clarke. Previously, sections in the C3 *Cyperus* grade + the C4 *Cyperus* section *Amabilis* were placed together in *Cyperus* subgenus *Pycnostachys* C.B. Clarke based on this shared inflorescence type *versus* the remaining sections in the C4 *Cyperus* clade, which are characterized by having spikes of spikelets. A notable difference with earlier studies is that remaining species in the C4 *Cyperus* clade form two well-supported clades (indicated as clade A and B; **Figures 4**, **6**, and **7**). One of the two groups of closely related species included in this study—i.e., the white-glumed *Cyperus* species or the *C. margaritaceus*-*C. niveus* complex (clade A)—is here reconstructed as sister to a clade (B) comprising the rest of the C4 *Cyperus* clade. Species of clade A had not been included in previous molecular studies. More research is needed to confirm that this is not a sampling artifact, however, although sampling in this study is limited, it adequately covers the range of morphological diversity observed in the C4 *Cyperus* clade, as it encompasses both species of C4 *Cyperus* s.s. (e.g., type species *C. esculentus* L.) and all 10 of the C4 segregate genera recognized by Goetghebeur (1998).

The position of *C. hystricoides* is unstable, being inferred either as sister to clade A or as part of the species-poor lineages in clade B (**Figures 4**, **6**, and **7**). This species was placed in *Rikliella* J. Raynal (Raynal, 1973) and later merged into *Lipocarpha* by Goetghebeur and Van den Borre (1989), who interpreted the inflorescence as a head of several spikes of spirally arranged single-flowered spikelets lacking a spikelet prophyll and glumes. However, an ontogenetic study (Bauters et al., 2014) showed that the inflorescence should be interpreted as a head of several spikelets with multiple spirally arranged flowers that have both a spikelet prophyll and glumes. With the new interpretation, the inflorescence type in *C. hystricoides* is similar to that of species previously placed in the C3 segregate genus *Kyllingiella* (now part of *Cyperus* sect. *Leucocephali*, incl. *Cyperus kyllingiella*; Larridon et al., 2011a), which is sister to the C4 *Cyperus* clade. This could provide morphological arguments for the placement of *C. hystricoides* among the species-poor lineages, away from the crown, of the C4 *Cyperus* clade.

Besides the *C. margaritaceus*-*C. niveus* complex, the other group of closely related species included in this study are seven species of *Cyperus* section *Kyllinga* (Rottb.) J. Kern (e.g., its type species *C. mindorensis*). Nodes within *Cyperus* sect. *Kyllinga* are well supported (**Figures 4**, **6**, and **7**), demonstrating the utility of the data obtained with both targeted sequencing kits to resolve low-level relationships in the C4 *Cyperus* clade. However, in the *C. margaritaceus*-*C. niveus* complex relationships between taxa are poorly supported although most morphologically defined taxa are retrieved as monophyletic, at least in the ASTRAL analyses (**Figures 4**, **6**, and **7**).

The results confirm the close relationship between *Cyperus laevigatus* (placed in the former segregate genus *Juncellus* C.B. Clarke) and *Cyperus polystachyos* (type species of the former segregate genus *Pycreus*) found in previous studies (e.g., Larridon et al., 2013; Semmouri et al., 2019). As in C3 *Cyperus* and C4 *Cyperus* s.s., the species previously placed in *Juncellus* and *Pycreus* have spikelets with multiple distichously arranged glumes each bearing a flower. However, in contrast to *Cyperus* s.s. with trigonous nutlets, *Juncellus* was recognized by dorsiventrally flattened nutlets, while in *Pycreus* nutlets are laterally compressed. In Cyperoideae, the development of the gynoecium from an annular primordium facilitates the shift in localization of stigma primordia (Vrijdaghs et al., 2011). Together with the decoupled development of the ovary and ovule (Reynders et al., 2012), this enables shifts between trigonous and dorsiventrally and laterally flattened nutlets in related species.

Our study is also the first to include all four species of the former segregate genus *Alinula* (*C. lipocarphioides*, *C. malawicus*, *Cyperus microaureus* Lye, *Cyperus subparadoxus* Kük.; **Figures 4**, **6**, and **7**). Earlier efforts to include all species in a Sanger sequencing study had failed due to degraded DNA extracted from herbarium specimens. This illustrates the advantage of targeted sequencing over Sanger sequencing for degraded DNA. *Alinula* sensu Goetghebeur (1998) is clearly polyphyletic (three groups). The first species to be published in *Alinula* was *C. lipocarphioides* by Raynal (1977) when he described the new genus. Later, another species, *C. malawicus*, was suggested to be its closest relative (Haines and Lye, 1983; Goetghebeur, 1986; Goetghebeur and Vorster, 1988). Our results confirm this close relationship. The species *Cyperus microaureus* was originally described in the segregate genus *Ascolepis*, but Goetghebeur (1977) relegated it to its own monotypic genus *Marisculus* Goetgh. because some of its inflorescence and spikelet characteristics are peculiar. Later, it was placed in *Alinula* (Goetghebeur and Vorster, 1988). In our results, the species appears sister to *Ascolepis* [represented by *C. ascocapensis* and *Cyperus eriocauloides* (Steud.) Bauters]. In his doctoral thesis, Vorster (1978) placed the fourth species, *Cyperus subparadoxus*, in a monotypic genus *Pseudolipocarpha* (not validly published) before moving it to *Alinula* (Goetghebeur and Vorster, 1988. It is here retrieved as a lineage separate from the other species formerly placed in *Alinula*.

## Conclusion

We show the utility of two targeted sequencing kits, the universal Angiosperms-353 kit and a Cyperaceae-specific kit, in resolving relationships in a fast-evolving and taxonomically complex plant lineage, i.e., the C4 *Cyperus* clade. The probes from both kits work well with the often-degraded DNA-template obtained from herbarium material and allow the resolution of long-standing questions in Cyperaceae systematics (e.g., concerning the former segregate genus *Alinula*), where Sanger sequencing was previously either unsuccessful or provided no resolution. Generally, high support is retrieved using data of either or both kits, but some issues remain for the shortest branches where either significant conflict in gene trees or lack of signal occurs as shown by quartet scores, and gene and sCF. Potentially, adding off-target flanking regions and retrieving off-target high-copy sequence data such as the plastid genome, may provide added resolution. Our results demonstrate that data generated with a family-specific kit do not necessarily have more power than those obtained with a universal kit, at least in the C4 *Cyperus* clade, but that data generated with different targeted sequencing kits can often be merged for downstream analyses. Moreover, our study contributes to the growing consensus that targeted sequencing data are a powerful tool in resolving rapid radiations. We encourage ongoing studies to use targeted sequencing in lieu of Sanger sequencing to investigate the evolutionary history of Cyperaceae. The short-term costs in the lab will surely be mediated by long-term savings, as data can be repurposed for population genetics and phylogenetics with no return to the lab to sequence *just one more locus*.

## Data Availability Statement

The data generated for this study can be found in Genbank SRA under Bioproject numbers PRJNA553989 (Cyperus Bioproject), PRJEB35281 (Cyperus Baits Bioproject) and PRJNA553631 (Schoenoplectus pungens – Carex Bioproject).

## Author Contributions

IL and TV contributed equally as first authors. IL, TV, and AZ conceived the project design. IL and MX performed the sampling. IL, TV, LP, GB, NE, IF, MH, EM, and OM were responsible for generating the sequence data. IL, TV, AZ, and LP conducted the bioinformatic and molecular evolutionary analyses and wrote the manuscript. WB, FF, and AH supervised the research. All authors read and commented on the manuscript.

## Funding

IL is supported by the B.A. Krukoff Fund for the Study of African Botany and a pilot study grant from the Royal Botanic Gardens, Kew. Phylogenomic work at Kew was funded by grants from the Calleva Foundation, the Sackler Trust and the Garfield Weston Foundation. The research of TV is supported by the Spanish Ministry of Economy and Competitiveness (project CGL2016-77401-P). The research of EM was supported by the Spanish Government, through Juan de la Cierva-Formación contract (FJCI-2017-32314). Carex NSF grant (NSF-DEB award #1255901) supported the lab work and sequencing at the Morton Arboretum. We acknowledge support of the publication fee by the CSIC Open Access Publication Support Initiative through its Unit of Information Resources for Research (URICI).

## Conflict of Interest

The authors declare that the research was conducted in the absence of any commercial or financial relationships that could be construed as a potential conflict of interest.
