# Supplementary material for: Tackling Rapid Radiations With Targeted Sequencing
Source: Front Plant Sci. 2020 Jan 9;10:1655. doi: 10.3389/fpls.2019.01655 (PMC6962237; doi:10.3389/fpls.2019.01655)
Supplement: Supplementary file 1 [file DataSheet_1.pdf]

96-well plate CTAB extraction

by Izzie Fairlie & Lisa Pokorny

Modified from Beck et al. 2012 (original protocol by Sandy Boles, formerly at Duke University) to maximize DNA yield for tissue from historical herbarium collections. This protocol permits simultaneous extraction of two 96-well plates (192 samples), which facilitates easy centrifuge balancing (downsizing is possible as long as tube strips are even in number).

Consumables (fisher scientific, UK)
– Sterile microtiter 1.2 ml tubes in racks (drill small holes through the base of a couple racks)
– Sterile microtiter tube plug strips x 2
– SPEX™ stainless steel grinding balls (reusable) and dispenser (provided with Geno/Grinder®)

Reagents
– Liquid nitrogen (approx. 1.5 L at ca. –196ºC)
– Freshly prepared 2x CTAB (65ºC) + BME buffer (0.2% if recent or silica dried or 0.4% if historical herbarium tissue or if rich in 2^{ary} metabolites)
– Sevag buffer = Chloroform:IAA (24:1)
– Isopropanol (100%, –20ºC)
– Ethanol (70%, freshly prepared)

A. **Plating tissue samples** (sampling two plates takes hours)

1. **Label** microtiter **tubes and plugs** to keep track of specimen identity and tube number.
2. Using dispenser, **add two** (Ø = 4mm) **grinding balls** (440C stainless-steel) to each microtiter tube in rack.
3. **Add** a small amount of **tissue** (ideally, no more than double the ball's height) from a target specimen into each microtiter tube (though the amount will vary by species).
4. **Cap the tubes** with the small plug-strip tabs (labelled) towards the top (A row) to make removal easier. Make sure the tubes are in 96-well **racks with holes drilled through the base**, which will enable the liquid nitrogen and hot water to filter out in later steps.

B. **Preparing CTAB+BME isolation buffer** (approx. 15 min)

5. **Set water bath to 65°C** (30 min before start) and ensure that the volume is appropriate for two 96-well racks (it should contain sufficient water to submerge the bottom half of the tubes, but not so much as to submerge the entire tube).
6. **Prepare fresh isolation buffer** by adding β-mercaptoethanol to **2x** stock **CTAB** buffer in a flask. Remember, add 0.2% BME if recent or silica dried tissue (optimal) **and 0.4% BME** (or more) if historical herbarium tissue (often doused in alcohol and/or mercury) or if lineage known to be rich in 2^{ary} metabolites (suboptimal). Keep warm on a hot plate when working with optimal tissue (not needed for suboptimal tissue).

C. **Grinding plant cells and DNA extraction with CTAB+BME buffer** (approx. 1 h)

7. First, **pour** a small amount (approx. 1.5 L) of **liquid nitrogen** (at ca. –196ºC) into a styrofoam cooler (i.e., shipping containers can be set aside and reused).
8. **Freeze** the **tissue by placing** the two (loaded and firmly capped with tube-plug strips) 96-well **racks** (with holes at the base) **in liquid nitrogen** (in styrofoam container). Metal centrifuge carriers are helpful for lowering and transferring the racks into and out of the liquid nitrogen, while wearing appropriate gear (i.e., cryogenic gloves).

9. After ca. 10–20 seconds **remove** the **frozen racks and** immediately **transfer** them **to** a **Geno/Grinder®** (e.g., 2010 SPEX) or similar tissue disruptor.
10. **Ensure** that the two racks are tightly clamped but no too much since the tubes could shatter. It is critical that the **clamp presses directly down on the strip tube caps** themselves: **<u>remove the 96-well rack lid</u>** (!!!). **Shake 1 min at 1500 strokes/min** (you can opt for lower speeds and/or a shorter shaking time to prevent shattering/cracking of tubes). After you remove your racks from the Geno/Grinder®, a short/quick spin can help remove excess plant tissue powder from tube caps.
11. **Gently remove strip caps** and deposit them downside up on a paper towel, being sure to note cap identity/orientation and taking care not to spread powdered tissue. Removing the caps will take some time, as the tubes will still be somewhat hardened from the liquid nitrogen step.
12. **Pour CTAB+BME buffer into** a plastic **pipetting reservoir and add 400 µl** to each sample **with a** p1200 **multichannel pipette** (or 200 µl twice with, i.e., a p300). This is best done **on the open lab bench**, as the powdered tissue can be drawn up by the suction of a fume hood. **Mix buffer with powdered tissue by pipetting up and down**. Try to get as much of the tissue in solution (a strong magnet can be used to dislodge the stainless steel balls to facilitate mixing), but don't worry if a small pellet in the bottom of the tube doesn't soak.
13. **Carefully replug the tubes**, taking care to add the caps to the corresponding strip tubes in the correct orientation.
14. Return all 8-tube strips to their racks (again the ones with holes at the base) and **incubate at 65°C in the water bath for <u>24 hours</u>** (if tissue suboptimal; an hour suffices for optimal tissue but it requires flicking tubes every 15 min). **Be sure to place a weight of some kind directly over the strip tube caps** to prevent these from opening during the incubation (upside down rack lid can be filled with a number of heavy items; a spare aluminum dry block works quite well; porcelain spot trays also do the trick).

D. **Removing proteins with chloroform:IAA** (= Sevag buffer) **and precipitating DNA** (ca. 1 h)

15. **Keeping the weight over lids**, **remove racks from hot water bath** and allow **to drain and cool down** for approx. 15 min **in a tub or tray**. While you wait, **label a new set of tubes and two sets of strip caps**.
16. Inside a fume hood, **pour** the required volume of (24:1) **chloroform:isoamyl alcohol into** a **<u>glass</u>** (staining) **dish** (and cap with glass lid to prevent evaporation) **for pipetting** (Sevag buffer dissolves plastic). Remove both the strip caps (can be thrown out) and the glass dispenser lid and **add** an equal volume (**400 µl**) **of** the (24:1) **chloroform:IAA** to the tubes with plant extract (incubated in CTAB+BME isolation buffer) **with a** p1200 **multichannel pipette** (or 200 µl twice with, i.e., a p300). **Cap tubes** securely **with <u>new</u> strip plugs** and add enough folded paper towel (to the inside of the rack lid) to fill the small gap between the tube caps and the rack lid. Alternatively, put folded paper towel over tube strip plugs and hold rack against upside down lid. **Mix by inverting** 25 to 50 times **each rack** (making sure to change rack orientation) **to produce an emulsion**.
17. **Centrifuge the two** (balanced!) **racks with lids for 10 min at 3700 rpm** in a plate centrifuge. Though 3700 rpm suffices, centrifuging could be done 5 min at a higher rpm.
18. **Transfer** (top) **aqueous phase to newly labeled tubes with** a **multichannel pipette**. Removing the top phase is difficult with a large volume multichannel pipette (i.e., p1200 or p300) but can be more easily done in two to three steps with a smaller p100 and by angling the tubes and going slowly (in this manner, it is possible to recover 200 to 250 µl without disturbing the interface). Take note of the volume of transferred aqueous phase for step #20 below. **Avoid disturbing or pipetting the interface** as it will have downstream consequences.
19. **Dispose of** tubes, ball bearings, plant extract, and chloroform:IAA **waste following regulations**.

20. **Add an equal volume of cold** (–20ºC) **isopropanol to the** transferred **aqueous phase**, after which you should see a white precipitate at the boundary.
21. **Cap tubes securely** (with <u>new</u> strip plugs), rack them, add a folded paper towel to the inside of the rack lid to fill the small gap between the tube caps and the lid, **and mix gently**, approx. 10 times, **by inverting each rack**. Alternatively, put folded paper towel over tube strip plugs and hold rack against upside down lid to invert and mix.
22. If tissue suboptimal, **precipitate** at -20°C **three to seven nights**. Overnight for optimal tissue.

E. **Washing, drying, and resuspending DNA** (approx. 2 h)

23. **Centrifuge** the (balanced!) **racks for 20 min at 3700 rpm** in a plate centrifuge. When the run ends a white, off-white, or even darkly colored pellet will often (though not necessarily) be visible at the bottom of many tubes.
24. **Remove caps and** deposit them downside up on a paper towel (being sure to note cap identity/orientation). Working with one 8-tube strip at a time, **pour off** the **isopropanol** (a deep glass dish or petri dish works well) and then remove the isopropanol remaining in the tube by dragging the tube strip along a clean paper towel. Be sure to drag quickly and in a straight line to avoid cross-contaminating tubes with isopropanol from adjacent tubes. Carefully monitor any visible pellets to ensure they are not being poured off. Note that if you wait too long after the centrifuge run ends many of the pellets detach from the tubes making it difficult to pour.
25. **Pour 70%** freshly prepared **ethanol into** a plastic **pipetting reservoir and add 500 µl** to all tubes **to wash pellets**, securely capping tubes, and gently vortexing each rack with a rack vortex, while pressuring the strip plugs with the upside down rack lid (or by carefully pipetting up and down). With sufficient vortexing most pellets will release from the tube bottoms and rotate in the ethanol.
26. Cap tubes securely, rack them, and **spin** the two (balanced!) **racks 15 minutes at 3700 rpm** in a plate centrifuge to secure pellets to tube bottoms.
27. **Remove caps and pour off the ethanol** as you did in step #24.
28. Do a **second 70% ethanol wash**. Repeat steps #25 through #27.
29. Do not re-cap the tubes (but do save them for later). Cover the racked, uncapped tube strips with a clean paper towel. You can **air-dry pellets** by either leaving them out overnight on a benchtop at room temperature, or by placing them **in an oven**/incubator **at** 40º to **60ºC for ca. an hour** (check every ten or so minutes and remove from oven/incubator once all ethanol has evaporated).
30. **Add 50-100 µl Milli-Q ultrapure water**, re-cap tubes (with saved lids), **and incubate racked tubes** for either ca. three hours at 37°C or **for 30 min at 60ºC in an oven**/incubator (in both cases **vortexing periodically** to assist with re-suspension).
31. Note that re-suspended DNA extracts may exhibit a range of colors from clear to black depending on age and quality of the extracted tissue. Proceed with your preferred downstream protocols like, further cleaning, DNA quantification, gelling/visualization, required dilutions, etc.

BEST OF LUCK!
