# Supplementary figures and images for: Tackling Rapid Radiations With Targeted Sequencing
Source: Front Plant Sci. 2020 Jan 9;10:1655. doi: 10.3389/fpls.2019.01655 (PMC6962237; doi:10.3389/fpls.2019.01655)

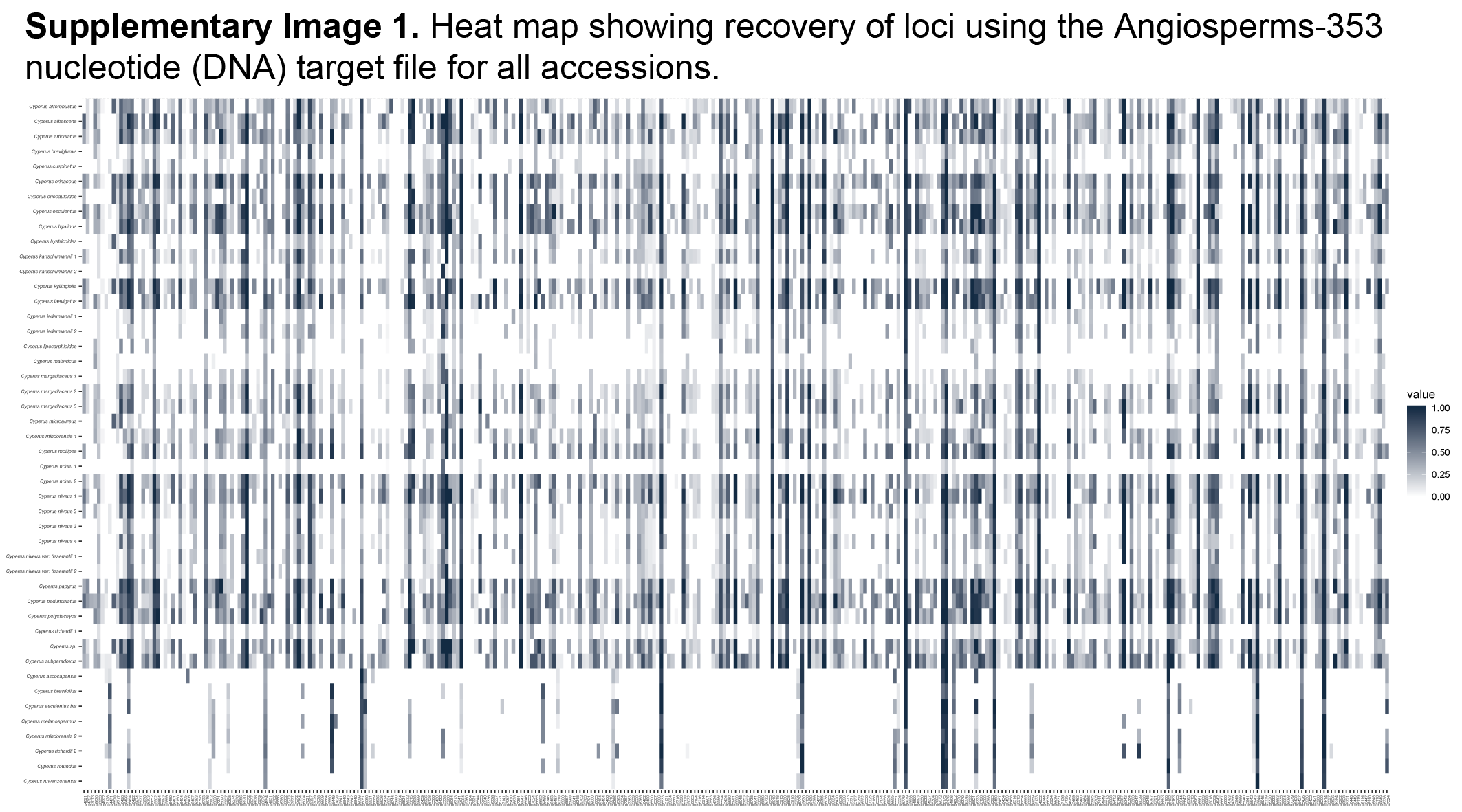

Supplement: Supplementary file 4 [file Image_1.tif]

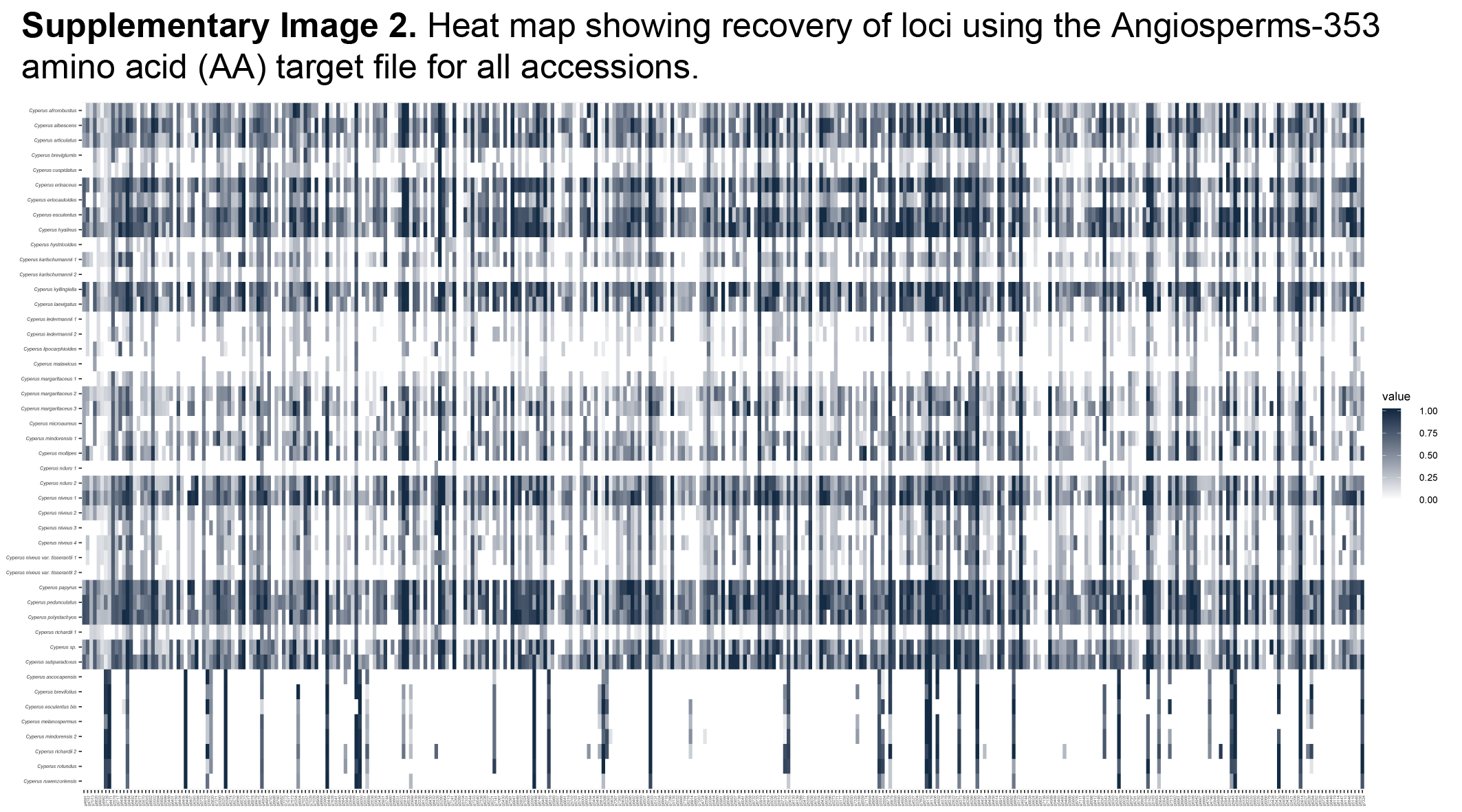

Supplement: Supplementary file 5 [file Image_2.tif]

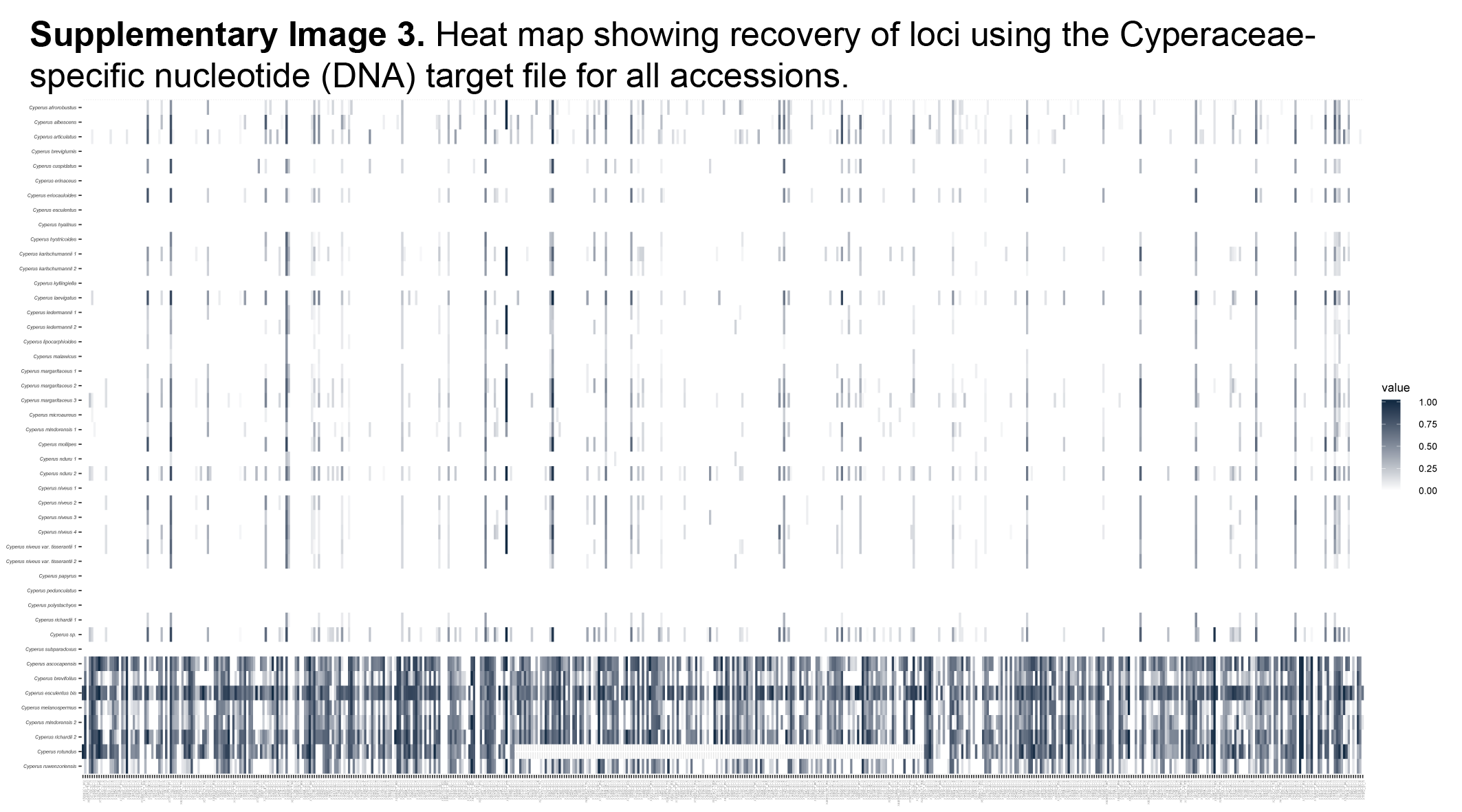

Supplement: Supplementary file 6 [file Image_3.tif]

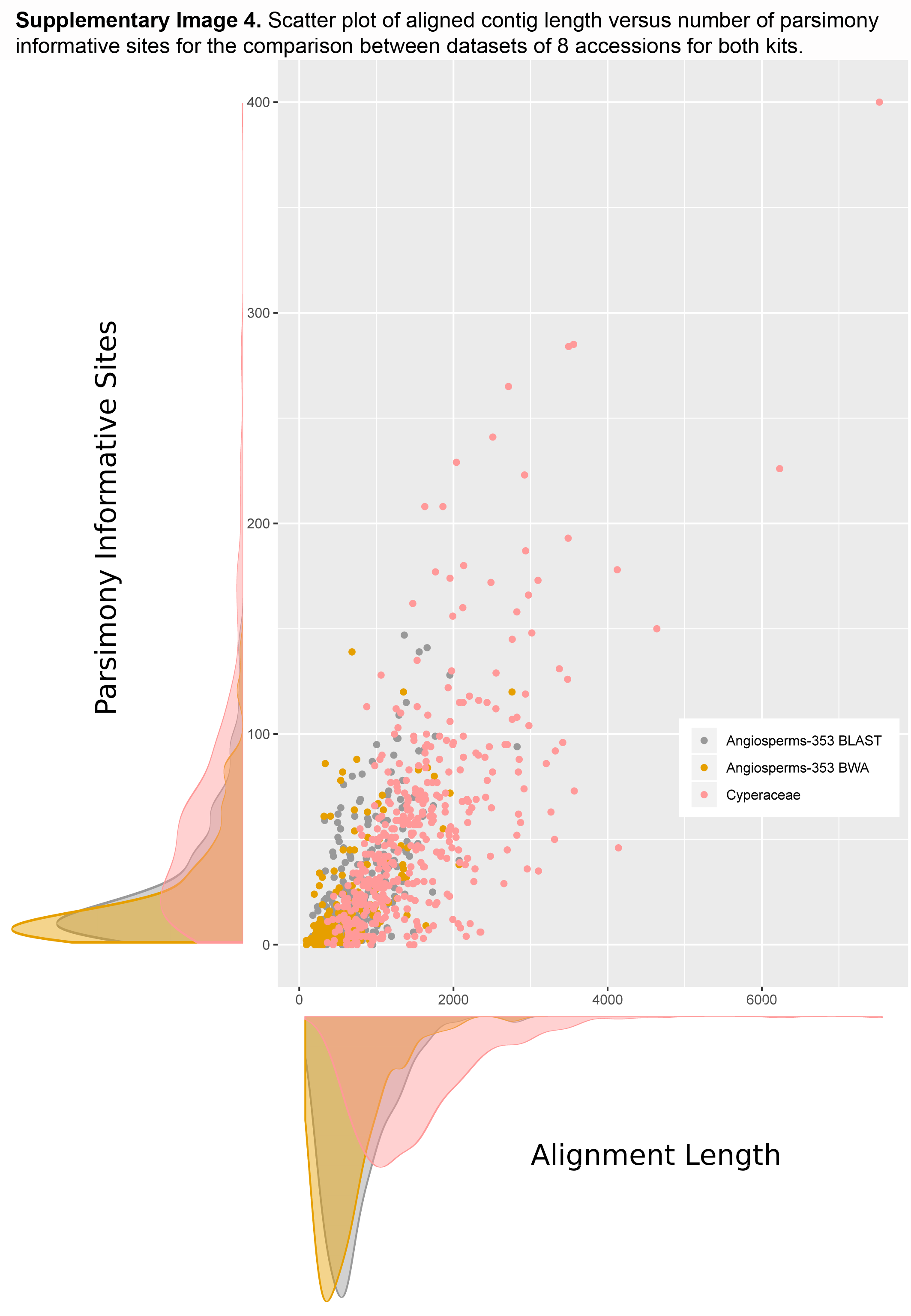

Supplement: Supplementary file 7 [file Image_4.tif]

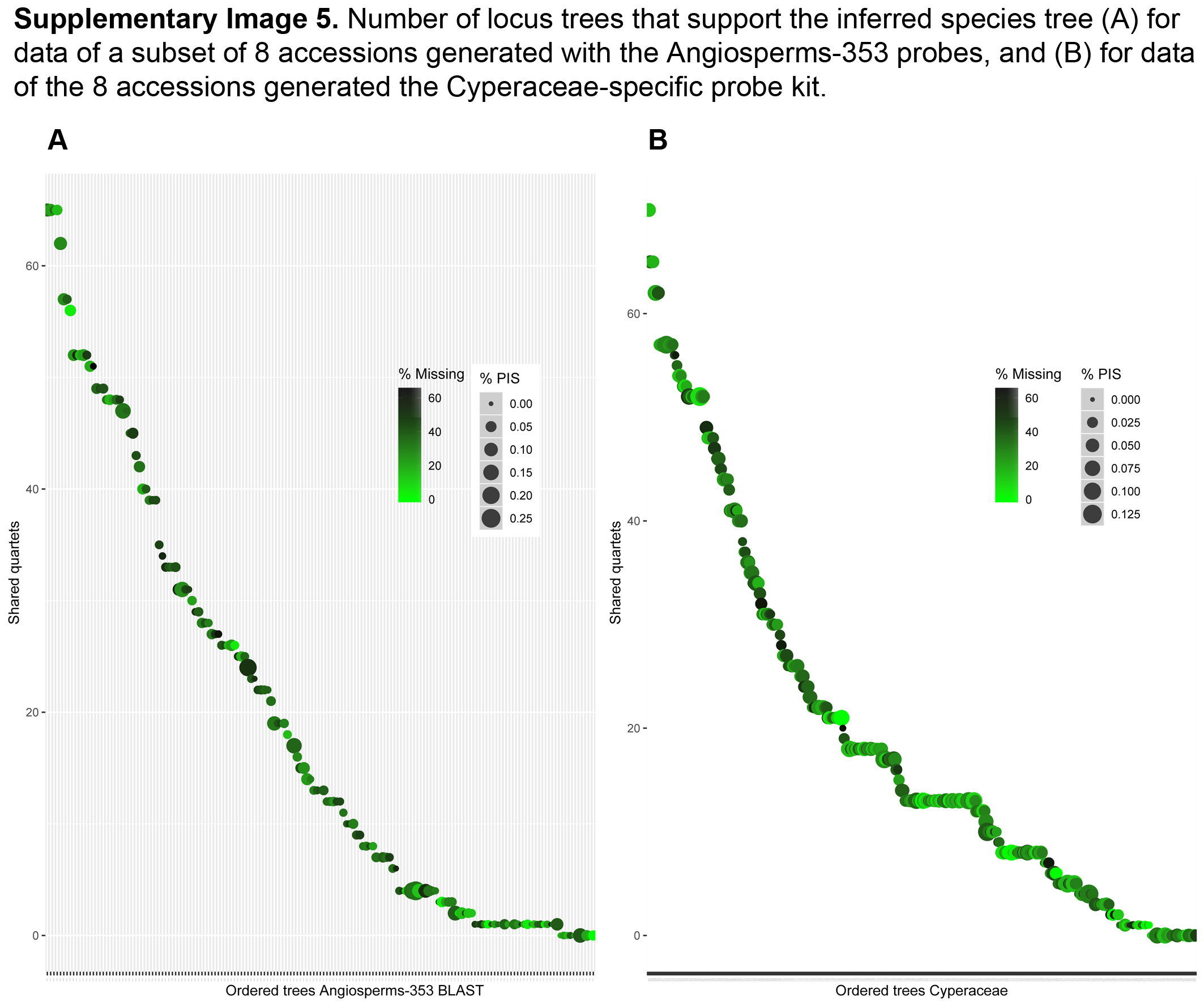

Supplement: Supplementary file 8 [file Image_5.tif]

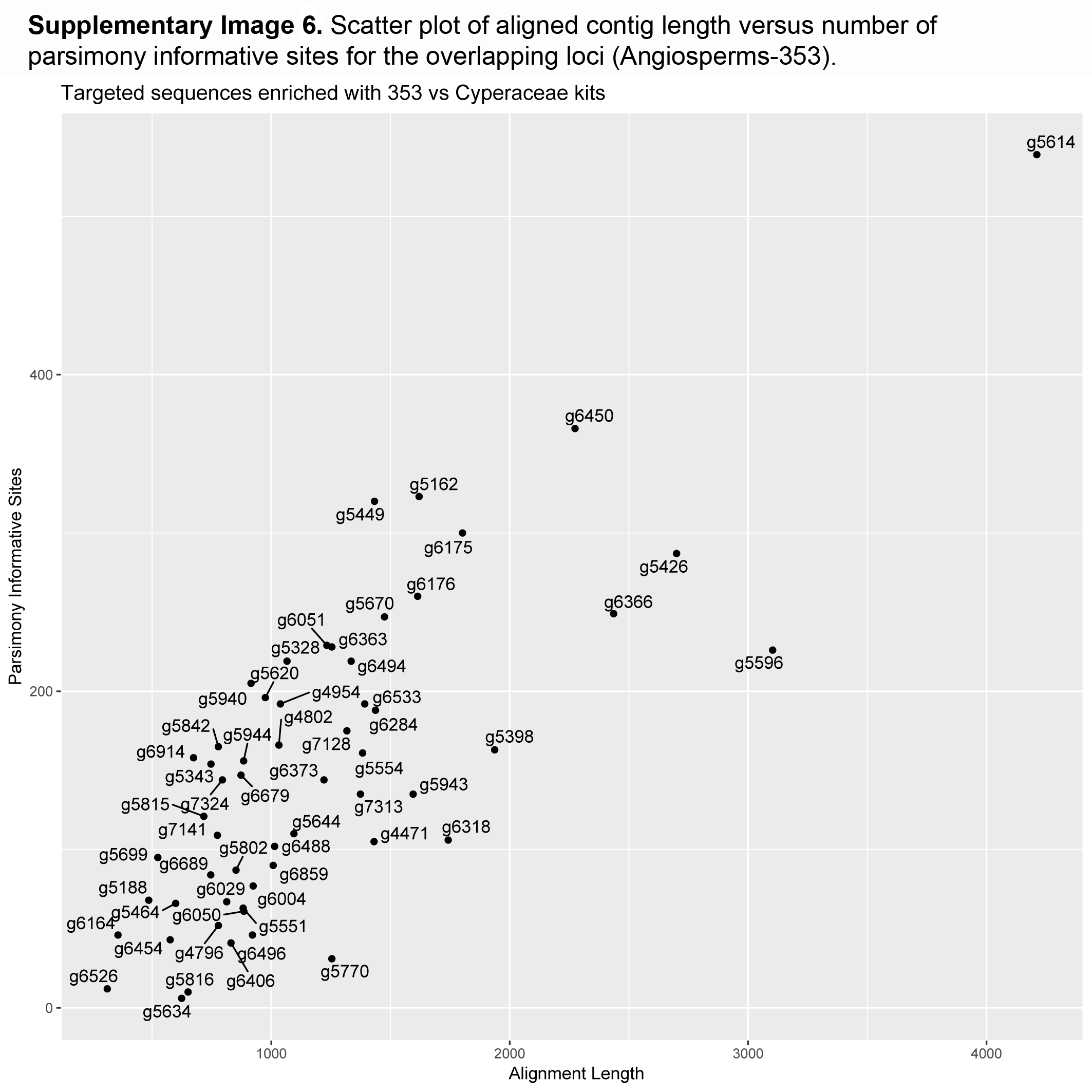

Supplement: Supplementary file 9 [file Image_6.tif]
