# Supplementary material for: Tackling Rapid Radiations With Targeted Sequencing
Source: Front Plant Sci. 2020 Jan 9;10:1655. doi: 10.3389/fpls.2019.01655 (PMC6962237; doi:10.3389/fpls.2019.01655)
Supplement: Supplementary file 10 [file Table_1.docx]

Supplementary Material

**Tackling hard polytomies with targeted sequencing**

**Isabel Larridon^1,2*†^, Tamara Villaverde^3,4,5*†^, Alexandre R. Zuntini^1^, Lisa Pokorny^1,3,6^, Grace E. Brewer^1^, Niroshini Epitawalage^1^, Isabel Fairlie^1,7^, Marlene Hahn^4^, Jan Kim^1^, Enrique Maguilla^4,8^, Olivier Maurin^1^, Martin Xanthos^1^, Andrew L. Hipp^4,5^, Félix Forest^1^, William J. Baker^1^**

*** Correspondence:**
Isabel Larridon
 [i.larridon@kew.org](mailto:i.larridon@kew.org) Tamara Villaverde
 [t.villaverde@rjb.csic.es](mailto:t.villaverde@rjb.csic.es)

**Supplementary Table 1.** Voucher information for the sampled accessions.

**A.** Sampling of accessions enriched with the Angiosperm-353 probes.

| **Name in tree** | **Accepted name** | **Voucher** | **DNA from** |
| --- | --- | --- | --- |
| *Cyperus afrorobustus** | *Cyperus afrorobustus* Lye | van der Burgt, X. 1803 (K) | Herbarium |
| *Cyperus albescens* | *Cyperus albescens* (Steud.) Larridon & Govaerts | Larridon et al. 2010-0013 (GENT) | Silica collection |
| *Cyperus articulatus* | *Cyperus articulatus* L. | Larridon I. 110 (K) | Herbarium |
| *Cyperus breviglumis** | *Cyperus breviglumis* Lye | Sanford, W.W. 5592 (K) | Herbarium |
| *Cyperus cuspidatus* | *Cyperus cuspidatus* Kunth | Bidgood, S. et al. 5855 (K) | Herbarium |
| *Cyperus erinaceus* | *Cyperus erinaceus (Ridl.) Kük.* | Faden et al. 96/358 (K) | Herbarium |
| *Cyperus eriocauloides** | *Cyperus eriocauloides* (Steud.) Bauters | J.J.FE. De Wilde 7042 (GENT) | Herbarium |
| *Cyperus esculentus** | *Cyperus esculentus* L. | Larridon et al. 2010-0130 (GENT) | Silica collection |
| *Cyperus hyalinus** | *Cyperus hyalinus Vahl* | Muasya, A.M. 2490 (EA) | Silica collection |
| *Cyperus hystricoides* | *Cyperus hystricoides* (B.Nord.) Bauters | Robinson, E.A. 3549 (K) | Herbarium |
| *Cyperus karlschumannii 1* | *Cyperus karlschumannii* C.B.Clarke | Assi 6513 (K) | Herbarium |
| *Cyperus karlschumannii 2* | *Cyperus karlschumannii* C.B.Clarke | Innes, R.R. 30840 (K) | Herbarium |
| *Cyperus kyllingiella* | *Cyperus kyllingiella* Larridon | Bidgood, S. et al. 6209 (K) | Herbarium |
| *Cyperus laevigatus* | *Cyperus laevigatus* L. | Muasya, A.M. 1042 (K, EA) | DNA bank 2471 |
| *Cyperus ledermannii 1* | *Cyperus ledermannii* (Kük.) S.S.Hooper | Keay, R.W.J. 25772 (K) | Herbarium |
| *Cyperus ledermannii 2* | *Cyperus ledermannii* (Kük.) S.S.Hooper | Keay, R.W.J. s.n. (K) | Herbarium |
| *Cyperus lipocarphioides* | *Cyperus lipocarphioides* (Kük.) Lye | Hooper, S.S. & Townsend 1863 (K) | Herbarium |
| *Cyperus malawicus* | *Cyperus malawicus* (J.Raynal) Lye | Robinson, E.A. 4440 (K) | Herbarium |
| *Cyperus margaritaceus 1* | *Cyperus margaritaceus* Vahl | Kornas, J. 2965 (K) | Herbarium |
| *Cyperus margaritaceus 2* | *Cyperus margaritaceus* Vahl | Bidault, E. 2482 (K) | Herbarium |
| *Cyperus margaritaceus 3* | *Cyperus margaritaceus* Vahl | Heath 2345 (K) | Herbarium |
| *Cyperus microaureus* | *Cyperus* *microaureus* Lye | Greenway, P.J. & Kanuri 13488 (K) | Herbarium |
| *Cyperus mindorensis 1** | *Cyperus mindorensis* (Steud.) Huygh | Lye, K.A., et al. 4285 (K) | Herbarium |
| *Cyperus mollipes* | *Cyperus mollipes* (C.B.Clarke) K.Schum. | Nijalingappa, B.H.M.  A1 (K) | DNA bank 21273 |
| *Cyperus sp.* | *Cyperus* sp. | Goyder, D. 8335 (K) | Herbarium |
| *Cyperus nduru 1* | *Cyperus nduru* Cherm. | Angus 1450 (K) | Herbarium |
| *Cyperus nduru 2* | *Cyperus nduru* Cherm. | Haba, P.K. 202 (K) | Herbarium |
| *Cyperus niveus var. tisserantii 1* | *Cyperus niveus* var. *tisserantii* (Cherm.) Lye | Friis, I. et al. 2403 (K) | Herbarium |
| *Cyperus niveus var. tisserantii 2* | *Cyperus niveus* var. *tisserantii* (Cherm.) Lye | Assi, A. 9805 (K) | Herbarium |
| *Cyperus niveus 1* | *Cyperus niveus* Retz. | Bauters, K. 2015-136 (GENT) | Silica collection |
| *Cyperus niveus 2* | *Cyperus niveus* Retz. | Friis, I. et al. 11272 (K) | Herbarium |
| *Cyperus niveus 3* | *Cyperus niveus* Retz. | Larridon, I. 119 (K) | Herbarium |
| *Cyperus niveus 4* | *Cyperus niveus* Retz. | Bidgood, S. et al. 6057 (K) | Herbarium |
| *Cyperus papyrus** | *Cyperus papyrus* L. | Larridon, I. 116 (K) | Silica collection |
| *Cyperus pedunculatus* | *Cyperus pedunculatus* (R.Br.) J.Kern | Faden et al. 96/48 (K) | Herbarium |
| *Cyperus polystachyos* | *Cyperus polystachyos* Rottb. | Larridon et al. 2010-0047 (GENT) | Silica collection |
| *Cyperus richardii 2** | *Cyperus richardii* Steud. | Deighton, F.C. 6075 (K) | Herbarium |
| *Cyperus subparadoxus* | *Cyperus subparadoxus* Kük. | Festo, L.; Luke, Q. 2578 (K) | Herbarium |

**B.** Sampling of accessions enriched with the Cyperaceae-specific probes.

| **Name in tree** | **Accepted name** | **Voucher** | **DNA from** |
| --- | --- | --- | --- |
| *Cyperus ascocapensis* | *Cyperus ascocapensis* Bauters | Larridon et al. 2010-0104 (GENT) | Silica collection |
| *Cyperus brevifolius* | *Cyperus brevifolius* (Rottb.) Hassk. | Larridon et al. 2010-0056 (GENT) | Silica collection |
| *Cyperus esculentus bis* | *Cyperus esculentus* L. | Larridon et al. 2010-0130 (GENT) | Silica collection |
| *Cyperus melanospermus* | *Cyperus melanospermus* (Nees) Suringar | Larridon et al. 2010-0095 (GENT) | Silica collection |
| *Cyperus mindorensis 2* | *Cyperus mindorensis* (Steud.) Huygh | Larridon et al. 2010-0224 (GENT) | Silica collection |
| *Cyperus richardii 2* | *Cyperus richardii* Steud. | Larridon et al. 2010-0045 (GENT) | Silica collection |
| *Cyperus rotundus* | *Cyperus rotundus* L. | Larridon et al. 2010-0001 (GENT) | Silica collection |
| *Cyperus ruwenzoriensis* | *Cyperus ruwenzoriensis* (C.B.Clarke) Huygh | Larridon et al. 2010-0233 (GENT) | Silica collection |
| *Schoenoplectus pungens* | *Schoenoplectus pungens* (M.Vahl) Palla | Martín-Bravo 86SMB15 (UPOS8687) | Silica collection |
