# Supplementary material for: Tackling Rapid Radiations With Targeted Sequencing
Source: Front Plant Sci. 2020 Jan 9;10:1655. doi: 10.3389/fpls.2019.01655 (PMC6962237; doi:10.3389/fpls.2019.01655)
Supplement: Supplementary file 12 [file Table_3.docx]

**Supplementary Table 3.** Potential paralogs removed from downstream analyses.

| **Name in tree** | **11952_g1_i1** | **46937C1_808** | **99251C1_878** | **19992_g1_i1** |
| --- | --- | --- | --- | --- |
| *Cyperus albescens* | 0 | 0 | 2 | 1 |
| *Cyperus ascocapensis* | 0 | 0 | 2 | 1 |
| *Cyperus erinaceus* | 0 | 0 | 3 | 1 |
| *Cyperus esculentus* | 0 | 0 | 2 | 1 |
| *Cyperus niveus 1* | 0 | 0 | 2 | 1 |
| *Cyperus nduru* *2* | 0 | 0 | 2 | 1 |
| *Cyperus rotundus* | 0 | 2 | 0 | 1 |
