# Supplementary material for: Tackling Rapid Radiations With Targeted Sequencing
Source: Front Plant Sci. 2020 Jan 9;10:1655. doi: 10.3389/fpls.2019.01655 (PMC6962237; doi:10.3389/fpls.2019.01655)
Supplement: Supplementary file 13 [file Table_4.docx]

**Supplementary Table 4.** The 57 overlapping loci targeted by the Angiosperms-353 and the Cyperaceae-specific probe kits.

| \| **Angiosperms-353** \| **Cyperaceae-specific** \| \| \| --- \| --- \| --- \| \| g7128 \| 15508_g2_i1 \| \| \| g4471 \| 21139_g1_i1 \| \| \| g6496 \| 12550_g1_i1 \| \| \| g6494 \| 21123_g1_i2 \| \| \| g6406 \| 17317_g1_i1 \| \| g5802 \| 5986_g1_i1 \| \| g5620 \| 20471_g1_i1 \| \| g5398 \| 10275_g1_i1 \| \| g4954 \| 20265_g1_i1 \| \| g6004 \| 20387_g1_i1 \| \| g5449 \| 10658_g1_i1 \| \| g5842 \| 18677_g1_i1 \| \| g6051 \| 19992_g1_i1 \| \| g6050 \| 16719_g1_i2 \| \| g6689 \| 16890_g1_i2 \| \| g5815 \| 21410_g6_i2 \| \| g5699 \| 17756_g1_i1 \| \| g5328 \| 21700_g1_i1 \| \| g7141 \| 13573_g1_i1 \| \| g6488 \| 10999_g1_i1 \| \| g6318 \| 17100_g1_i1 \| \| g6164 \| 21207_g1_i2 \| \| g5816 \| 18633_g1_i2 \| \| g6366 \| 21758_g1_i1 \| \| g6363 \| 13883_g1_i1 \| \| g5634 \| 21298_g1_i1 \| \| g6533 \| 19222_g1_i2 \| \| g6914 \| 20195_g1_i2 \| \| g5596 \| 18911_g1_i1 \| \| g5770 \| 18436_g1_i1 \| \| g5670 \| 19660_g1_i2 \| \| g5188 \| 19690_g1_i4 \| \| g5551 \| 18796_g1_i1 \| \| g5554 \| 13267_g1_i1 \| \| g6679 \| 19929_g3_i6 \| \| g6175 \| 20511_g1_i2 \| \| g6176 \| 1789_g1_i1 \| \| g4802 \| 11097_g1_i1 \| \| g6373 \| 15967_g1_i1 \| \| g6450 \| 21815_g2_i1 \| \| g6454 \| 22125_g1_i10 \| \| g6859 \| 17589_g1_i1 \| \| g5464 \| 18724_g2_i1 \| \| g5343 \| 14555_g1_i2 \| \| g6029 \| 16904_g1_i2 \| \| g5162 \| 9505_g1_i1 \| \| g5644 \| 19345_g1_i1 \| \| g7313 \| 13423_g1_i2 \| \| g4796 \| 12214_g1_i3 \| \| g5940 \| 21757_g1_i4 \| \| g5943 \| 19957_g1_i1 \| \| g5944 \| 35289_g1_i1 \| \| g5426 \| 21594_g7_i1 \| \| g5614 \| 17232_g1_i1 \| \| g6526 \| 37626_g1_i1 \| \| g6284 \| 20202_g1_i2 \| \| g7324 \| 9508_g1_i1 \| |  |
| --- | --- | --- | --- | --- | --- | --- | --- | --- | --- | --- | --- | --- | --- | --- | --- | --- | --- | --- | --- | --- | --- | --- | --- | --- | --- | --- | --- | --- | --- | --- | --- | --- | --- | --- | --- | --- | --- | --- | --- | --- | --- | --- | --- | --- | --- | --- | --- | --- | --- | --- | --- | --- | --- | --- | --- | --- | --- | --- | --- | --- | --- | --- | --- | --- | --- | --- | --- | --- | --- | --- | --- | --- | --- | --- | --- | --- | --- | --- | --- | --- | --- | --- | --- | --- | --- | --- | --- | --- | --- | --- | --- | --- | --- | --- | --- | --- | --- | --- | --- | --- | --- | --- | --- | --- | --- | --- | --- | --- | --- | --- | --- | --- | --- | --- | --- | --- | --- | --- | --- | --- | --- | --- |
